# Avoidance of apoptotic death via a hyperploid salvage survival pathway after platinum treatment in high grade serous carcinoma cell line models

**DOI:** 10.18632/oncotarget.27330

**Published:** 2019-11-19

**Authors:** Tony Yeung, Oliver Fung, Mikhail Bashkurov, Arian Khandani, Omar Subedar, Alexandra Wudwud, Patricia Shaw, Blaise Clarke, John Bartlett, Robert Rottapel, Andras Kapus

**Affiliations:** ^1^St. Michael’s Hospital, Keenan Research Center, Toronto, Canada; ^2^Network Biology Collaborative Centre, Toronto, Canada; ^3^Flow and Mass Cytometry Facility, Hospital for Sick Children, Toronto, Canada; ^4^Princess Margaret Cancer Center at the University Health Network, Toronto, Canada; ^5^Ontario Institute for Cancer Research, University of Toronto, Toronto, Canada; ^6^Department of Medicine, University of Toronto, Toronto, Canada; ^7^Division of Rheumatology, St. Michael’s Hospital, Toronto, Canada; ^8^Department of Immunology, University of Toronto, Toronto, Canada; ^9^Department of Medical Biophysics, University of Toronto, Toronto, Canada

**Keywords:** hyperploid genome, apoptosis, platinum chemotherapy, cell cycle checkpoint, ovarian cancer

## Abstract

The alkylating agent platinum is first-line chemotherapy treatment for high-grade serous carcinomas (HGSC) of tubal-ovarian origin. Platinum compounds cause DNA damage and induce apoptotic cell death in the bulk tumor population. However, subpopulations of tumor cells may exhibit diverging behaviors from the bulk tumor due to an alternate stress response that diverts tumor cells from apoptotic death. In this study, we identified a salvage survival pathway in which G2-arrested tumor cells bypassed apoptosis and progressed through aberrant mitotic events to then emerge as a distinct subpopulation of viable large hyperploid cells but with uncertain long-term propagation potential. Platinum-induced large hyperploid cells were flow sorted and showed rare regrowth capacity as compared to their more proficiently regenerating non-hyperploid counterparts. However, detailed time-lapse microscopy provided direct evidence that these hyperploid cells were mitotically active and could divide successfully to produce viable daughter cells. The *hyperploid survival response* was observed across different cell lines and utilization of this survival pathway was dependent on the strength of the G2-M checkpoint. Conceivably, this salvage survival strategy may contribute to increased genomic diversity of the regenerating tumor cell line through a coupled hyperploidization and de-polyploidization process that may be relevant for drug resistance.

## INTRODUCTION

High grade serous carcinoma (HGSC) of tubal-ovarian origin is an aggressive epithelial tumor with poor survival outcomes [1]. Platinum drugs are used as part of first-line therapy and exert genotoxic effects to tumor cells [2]. Major cellular responses to platinum treatment include initiation of the DNA damage response and the activation of the appropriate cell cycle checkpoint [3]. For tumor cells lacking a functional G1-S checkpoint such as in high grade serous carcinoma where the retinoblastoma (RB) and p53 pathways are perturbed, an arrest in cell cycle at the G2 phase is critical for DNA damage repair [4–6].

The serine/threonine kinase CHEK1 (CHK1) is an important regulator of the G2-M checkpoint downstream of the ataxia telangiectasia and Rad3-related protein (ATR) kinase, activated by DNA damage from genotoxic agents or replicative stresses [7, 8]. ATR phosphorylates serine 317 and 345 on CHK1, resulting in CHK1 conformational changes and enhanced kinase activity [9, 10]. Upon activation, CHK1 phosphorylates members of the CDC25 family of phosphatases, leading to their inactivation and the subsequent maintenance of the mitotic cyclin complex (Cyclin B:CDC2) in its inactive state [11]. By stalling cells at the G2 phase, proper DNA damage repair can be performed to allow cell survival whereas irreparable double strand breaks would lead to apoptotic death [12].

While this well-established DNA damage response operates at a global level for the bulk tumor, there may be unique subpopulational behaviors that divert from this canonical stress response. An alternate pathway has been described wherein tumor cells may prematurely exit the G2-M checkpoint and progress through mitosis. The entry to mitosis with residual DNA damage often results in mitotic catastrophe-mediated cell death but occasional tumor cells may circumvent mitotic death by failure to complete telekinesis and revert back to a single cell state termed “mitotic slippage” [13]. Subsequent to slippage, the genome may undergo endo-reduplication to generate a subpopulation of viable polyploid cells [13, 14].

The mitotic progression and subsequent polyploidization process may explain the documented nuclear morphologic changes observed in patient tumors after neo-adjuvant chemotherapy treatment. While chemotherapy treatment induces apoptotic cell death in the tumor, there are additional morphologic changes within the treated tumor field that suggest alternate mode of cell death or occasional survival via non-canonical response to treatment. Several previous reports showed that residual tumor cells from post treatment resection specimens of high grade serous carcinomas developed nuclear enlargement and bizarre nuclear morphology [15–17]. We surmised these residual tumor cells bear the characteristics of hyperploidy. However, neither the DNA ploidy status of these tumor cells, nor the mechanism of their derivation and biological significance has been elucidated.

Part of the challenge with the evaluation and functional interrogation of these unusual hyperploid tumor cells originates from the difficulty to effectively follow the generation and the cell fate of this rare, dynamic subpopulation. We recently refined an image cytometry analytical approach that enables the rapid evaluation of the cell cycle and DNA ploidy state at the single cell level using the DNA-binding Hoechst-33342 dye (Yeung et al. manuscript in preparation). The cell cycle and DNA ploidy status are then additionally correlated with markers of key cellular programs such as the DNA damage response and G2-M cell cycle checkpoint pathway via immunofluorescence staining. As this approach analyzes all tumor cells within a population at the single cell level, distinct subpopulational responses to treatment within the bulk tumor cell line can be revealed.

Using this refined technical approach, here we demonstrate that platinum treatment induces nuclear enlargement in the surviving cell population of several HGSC cell line models, and this *in-vitro* observation resembles closely to the morphologic changes seen *in-vivo* in patient tumors after neoadjuvant chemotherapy treatment. We further show mechanistically that the nuclear enlargement phenomenon is a morphologic manifestation of the deregulation of the G2-M checkpoint, through which a subpopulation of tumor cell survivors transitioned to an intermediate hyperploid state. The significance of the hyperploid subpopulation is assessed by sorting and serial dilution plating experiments, which show rare colony outgrowths from a moderately enriched hyperploid fraction. More detailed time-lapse analysis illustrates the capacity for successful mitosis and cellular division by the hyperploid subpopulation, highlighting the possibility of progenies from this unique subpopulation to reside within the regenerating tumor. We propose the hyperploid pathway as a salvage survival strategy for the individual tumor cell otherwise facing apoptotic death, and potentially as a mechanism to maintain intra-tumoral diversity for the bulk tumor during chemotherapy treatment.

## RESULTS

### Platinum treatment resulted in apoptotic and necrotic cell death in the bulk tumor cell population

**Figure 1:**
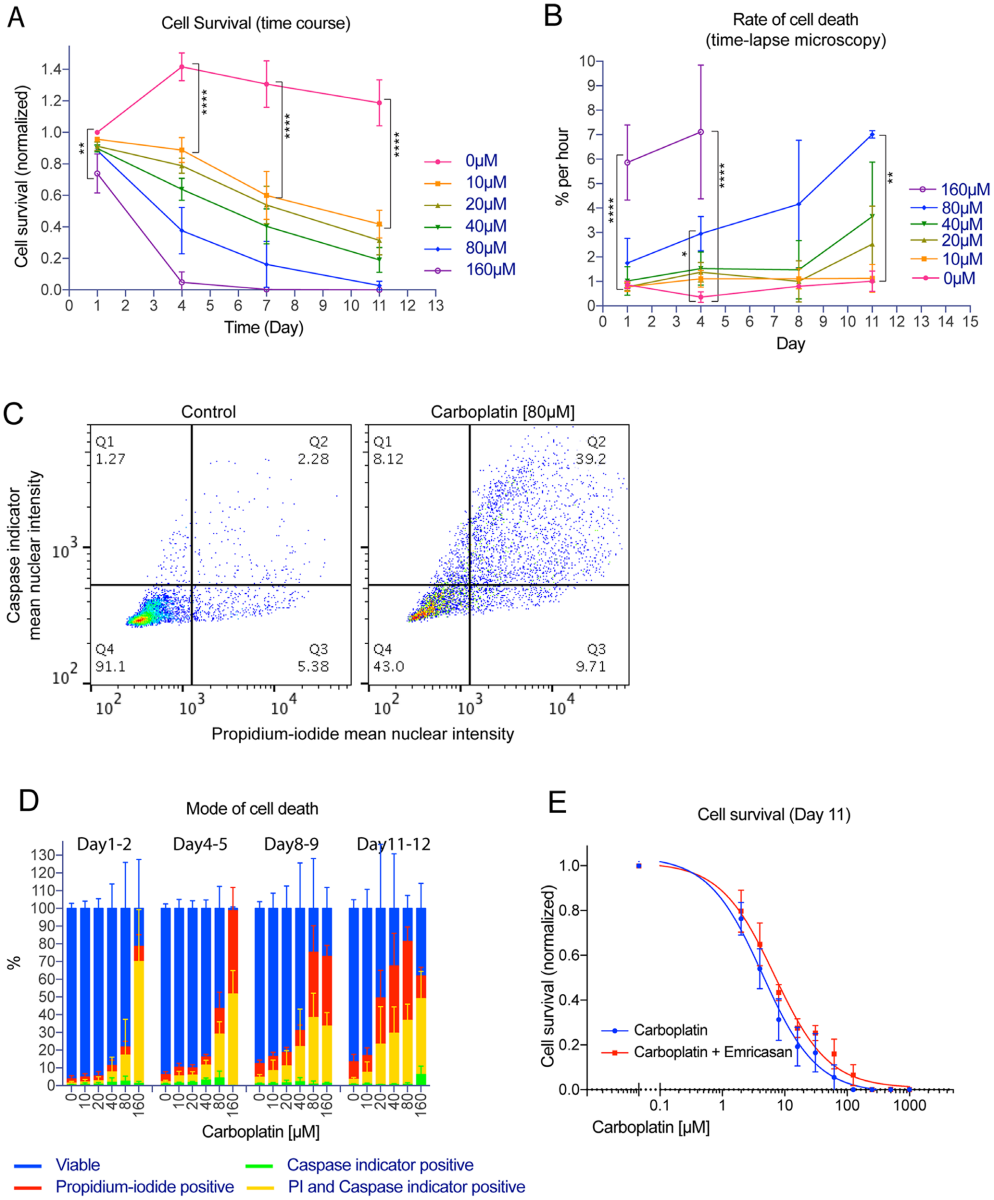
Platinum treatment resulted in apoptotic and necrotic cell death in the bulk tumor cell population. Time course, dose response or live imaging experiment where adherent OVCAR3 cells maintained within 96-well plates were treated with carboplatin from 0 µM and up to 1000 µM for 24 hrs on day 0, followed by drug removal. For a typical time course experiment, a set of four similarly prepared 96-well plates were all treated on day 0 and then one plate within the set would be fixed on day 1, 4, 7 or 11. (**A**) Time course of cell survival showing propidium-iodide excluded, Hoechst-stained nuclei count at 1, 4, 7 or 11 days after carboplatin treatment with the indicated concentration. Data points were mean nuclei count per well indicating cell survival (normalized to control of day 1) +/– standard deviations from 3 independent experiments. Nuclei analyzed ranged from 0 to about 20000 nuclei per well. ^**^ *p* < 0.01 and ^****^ *p* < 0.0001 using two-way ANOVA analysis with Bonferroni’s correction to demonstrate statistically significant differences between the 0 µM and the indicated carboplatin concentration. (**B**) Rate of cell death by time-lapse microscopy analysis from all causes after carboplatin treatment with the indicated concentration. Data points were mean percentage of cell death (based on the starting cell count) per hour +/– standard deviations from 3 independent experiments. Nuclei count ranged from 0 to 10000 nuclei per well. All death events ranged from 50 to 6000 events per well over a 10 to 24 hour-interval. ^*^ *p* < 0.05, ^**^ *p* < 0.01, and ^****^ *p* < 0.0001 using two-way ANOVA analysis with Bonferroni’s correction to demonstrate statistically significant differences between the 0 µM and the indicated carboplatin concentration. (**C**) Typical gating to quantify propidium-iodide positive and/or caspase indicator positive cells in the control (left; 6390 nuclei) or carboplatin treated condition (right; 4284 nuclei). Propidium-iodide and caspase indicator (CellEvent caspase 3/7 green indicator) positivity were defined on the plot of mean nuclear PI intensity vs. mean nuclear caspase indicator intensity to identify cells separated from the main viable cluster in the control sample. (**D**) Breakdown of percent viable, early apoptotic cell death (caspase-indicator positive), late apoptotic cell death (PI and caspase-indicator positive), or necrotic cell death (propidium-iodide positive) between day 1–2, 4–5, 8–9, 11–12. Data bars were mean percentage +/– standard deviations from 3 independent experiments. Nuclei analyzed ranged from 0 to 10000 nuclei per well. (**E**) Cell survival showing propidium-iodide excluded, Hoechst-stained nuclei count on day 11 after treatment with carboplatin on day 0 and either vehicle or the pan-caspase inhibitor Emricasan (2 µM or 5 µM). Emricasan was applied on day 0 or day 1 and then maintained and refreshed until the end of the experiment. Data points were mean nuclei count per well indicating cell survival (normalized to the 0 µM carboplatin condition) +/– standard deviations from 3 independent experiments. Nuclei analyzed ranged from 0 to about 17000 nuclei per well. The IC50 values from the two different conditions (4.6 +/– 0.5 µM vs. 7.0 +/– 0.8 µM) were not statistically significantly different (*p* = 0.4 using unpaired two-tailed *t*-test).

To evaluate the cytotoxicity effect of platinum treatment, cell survival was quantified by counts of propidium-iodide excluded nuclei. Adherent OVCAR3 cells were treated with 10 µM to 160 µM carboplatin on day 0 for 24 hours, followed by removal of the drug. Surviving cells on day 1, 4, 7 or 11 were then processed for propidium-iodide and Hoechst 33342 staining for viability analysis. Figure 1A shows a time and concentration-dependent cytotoxic effect of the carboplatin treatment, with half reduction of cell survival at 10 µM and complete elimination of the tumor cell population by the 160 µM treatment. Further time-lapse microscopy analysis of live Hoechst-stained OVCAR3 cells after similar carboplatin treatment revealed concentration-dependent increase in the rate of cell death (Figure 1B). Substantial level of cell death was already observed following the 80 µM to 160 µM carboplatin treatment on day 1 whereas it was more gradual with the 10 µM to 40 µM treatment (Figure 1B).

To provide an overview of the major modes of cell death, the fraction of cells undergoing apoptotic or necrotic cell death was evaluated using a caspase 3/7 activity indicator or the membrane impermeable dye propidium-iodide, respectively. Caspase 3/7 positive nuclei without propidium iodide indicate early apoptosis whereas caspase 3/7 and propidium iodide positive nuclei indicate late apoptosis. Propidium iodide staining alone indicates necrosis. While there was a small baseline level of apoptotic and/or necrotic cell death in the control condition (Figure 1C, left panel), carboplatin treatment induced substantial increase in the fraction of cells undergoing apoptosis (early or late) or necrosis (Figure 1C right panel), in a concentration and time-dependent manner (Figure 1D). Most of the platinum-mediated cell death was caspase 3/7 positive, suggesting the role of the apoptotic cell death program. However, up to half of the cell death at the late time point (day 11–12) was independent of apoptosis, suggesting the presence of other modes of cell death including necrosis (Figure 1D).

To further assess the role of apoptosis in mediating cell death by carboplatin, cells were treated with carboplatin and either vehicle or the pan-caspase inhibitor Emricasan (Figure 1E). The efficacy of Emricasan to inhibit apoptosis was observed in cells that exhibited less caspase 3/7 staining, and demonstrated a shift toward necrotic cell death (Supplementary Figure 1A). The IC50 value for carboplatin cytotoxicity was 4.6 +/– 0.5 µM whereas the inhibition of apoptosis by Emricasan showed no statistically significant enhancement of the IC50 value (7.0 +/– 0.8 µM; *p* = 0.4). The lack of enhanced survival with caspase inhibition suggests that the apoptotic pathway may not be the only dominant mode of cell death following platinum treatment at late time points.

### Platinum-induced nuclear enlargement as a distinct cellular response among surviving tumor cells at intermediate to late time points

**Figure 2:**
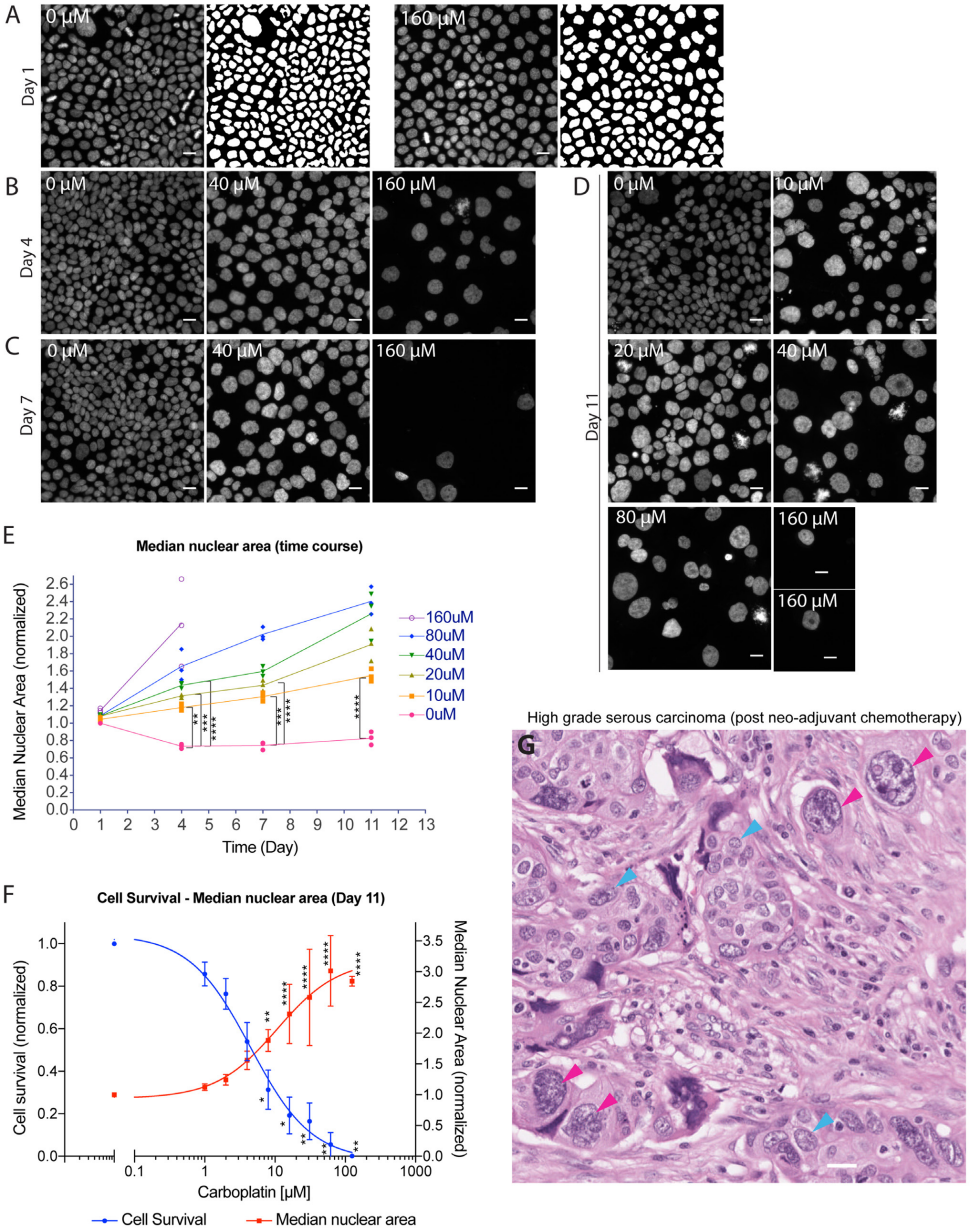
Platinum-induced nuclear enlargement as a distinct cellular response among surviving tumor cells at intermediate to late time points. Time course experiment where adherent OVCAR3 cells maintained within a set of four similarly prepared 96-well plates were all treated with 0 µM to 160 µM carboplatin for 24 hrs on day 0, followed by drug removal. One plate from the set of four was then fixed on day 1, 4, 7 or 11, respectively. (**A**–**D**) Hoechst 33342-stained nuclei, and where presented, the corresponding nuclear masks as identified by automated image analysis software for control vs. carboplatin treated cells on day 1 (A), day 4 (B), day 7 (C) or day 11 (D). Scale bar = 20 µm. (**E**) Time course of median nuclear area for control vs. carboplatin treated cells. Data points were median nuclear area from each individual experiment and the connected line passed through the average of the three biological replicates. Median nuclear area assessed from well with at least 5 to about 20000 nuclei. ^**^ *p* < 0.01, ^***^ *p* < 0.001 and ^****^ *p* < 0.0001 using two-way ANOVA analysis with Bonferroni’s correction to demonstrate statistically significant differences between 0 µM and the indicated carboplatin concentration. (**F**) Carboplatin concentration-dependent effect on cell survival (blue curve) or median nuclear area (red curve) on day 11 is shown. Data points were mean nuclei count per well indicating cell survival (propidium-iodide excluded; normalized to 0 µM condition) or average of median nuclear area (normalized to 0 µM condition) +/– standard deviations from 3 independent experiments. Nuclei analyzed ranged from 1 to about 16000 nuclei per well. Median nuclear area assessed from well with at least 5 nuclei. ^*^ *p* < 0.05, ^**^ *p* < 0.01 and ^****^ *p* < 0.0001 using two-way ANOVA analysis with Bonferroni’s correction to demonstrate statistically significant differences between 0 µM and the indicated carboplatin concentration. (**G**) Representative image of high grade serous carcinoma removed from the patient after neo-adjuvant chemotherapy treatment. Red arrowheads indicate several large tumor cells with marked nuclear enlargement due to the chemotherapy treatment whereas blue arrowheads indicate typical baseline morphology of HGSC tumor cells. Scale bar = 20 µm.

Apoptotic cell death is characterized by cell shrinkage, membrane blebbing, chromatin condensation, and nuclear fragmentation whereas necrosis is accompanied by organellar swelling and early disruption of membrane integrity. While the morphologic changes associated with apoptosis or necrosis reflected the bulk tumor cytotoxic response, we observed separate and distinct cellular response among the surviving tumor cells after platinum treatment. In contrast to dying cells, a portion of the surviving cells exhibited nuclear enlargement and bizarre nuclear shape changes. To systematically and quantitatively assess this nuclear response to platinum treatment, we applied a wide range of carboplatin concentrations to several representative ovarian cancer cell lines and monitored the change in nuclear area at different days after treatment. OVCAR3 cells were treated with 0 µM to 160 µM carboplatin as described earlier and surviving cells on day 1 (Figure 2A), 4 (Figure 2B), 7 (Figure 2C) or 11 (Figure 2D) were then fixed and processed for staining with the DNA-binding dye Hoechst 33342 to assess for changes in nuclear area. While carboplatin treatment did not cause significant nuclear area change after 24 hour, there was significant increase in nuclear area in the surviving cells starting from day 4 in a time and concentration-dependent manner (Figure 2E). The nuclear area, as determined only from the propidium-iodide excluded tumor cells, increased 1.6, 2.3 and 3.0-fold at the IC50 (5 µM), IC80 (16 µM), and IC95 (62 µM) level, respectively (Figure 2F). Similar nuclear enlargement was also observed in the TOV3133G (Supplementary Figure 1B) and PEA1 (Supplementary Figure 1C) ovarian cancer cell lines, along with a SV40 large T-antigen transformed lung epithelial cell line 16HBE (Supplementary Figure 1D). In contrast, a non-transformed fibroblast cell line HGF-1 did not show nuclear enlargement (Supplementary Figure 1E).

More importantly, these *in-vitro* derived large surviving tumor cells resembled closely those identified in a subset of patient tumors of high grade serous carcinoma (HGSC) after neo-adjuvant chemotherapy treatment (Figure 2G). From a cohort of ten resection specimens of HGSC obtained after chemotherapy treatment, five cases were found to exhibit these unusual nuclear morphologic changes (Supplementary Figure 2). While there was a baseline level of nuclear pleomorphism and nuclear area variation associated with high grade serous carcinoma, chemotherapy treatment induced substantial nuclear enlargement in a subpopulation of residual tumor cells beyond the usual variation. This was consistent with prior observations by McCluggage et al. where 9 of 9 matched HGSC showed increase in mean nuclear area of residual tumor cells in the post-treatment sample when compared to the pre-treatment sample (median increase = 1.3 fold; 1.1–1.9 at the 25% or 75% percentile) [17]. Based on the similarity between the experimental *in-vitro* and the *in-vivo* nuclear phenotype, we pursued further mechanistic analysis of these unusual large tumor cells induced by platinum treatment.

### Nuclear area and DNA ploidy analysis revealed G2-arrest of tumor cells after platinum treatment followed by the *de novo* generation of a large hyperploid subpopulation

Surviving cells following platinum treatment were likely arrested at the G2 phase for DNA-damage repair and we hypothesized that the nuclear area change might be reflective of the change in cell cycle status. We recently developed a refined image cytometry analytical approach for cell cycle status assessment based on a previous report [18]. Using this approach (Yeung et al. manuscript in preparation), the DNA ploidy of OVCAR3 cells was identified based on total Hoechst DNA content as assessed from individual nuclei on confocal images detected using an automated nuclear segmentation algorithm. The baseline ploidy of the OVCAR3 cell line was reported to be in the sub- to near-triploid range [19]. Therefore, the triploid status (3N) would correspond to the cell cycle phase Go/G1, whereas the hexaploid status (6N) would correspond to the G2 phase. In addition to Hoechst staining, the use of EdU (5-ethynyl-2′-deoxyuridine) pulse labeling enabled the identification of cells in the S-phase (Supplementary Figure 3). Gating to yield cells in the G1 (/Go), S and G2 phase of the cell cycle based on the Hoechst DNA ploidy and EdU incorporation level was then performed. Importantly, we further defined a threshold level to the right of the G2 gated region beyond which cells with supra G2 or hyperploid Hoechst DNA content (>6N) could be identified (Supplementary Figure 3). The image cytometry analysis provides the nuclear area of individual tumor cells in conjunction with their associated DNA ploidy status, thereby establishing the relationship between nuclear area and DNA ploidy/cell cycle.

**Figure 3:**
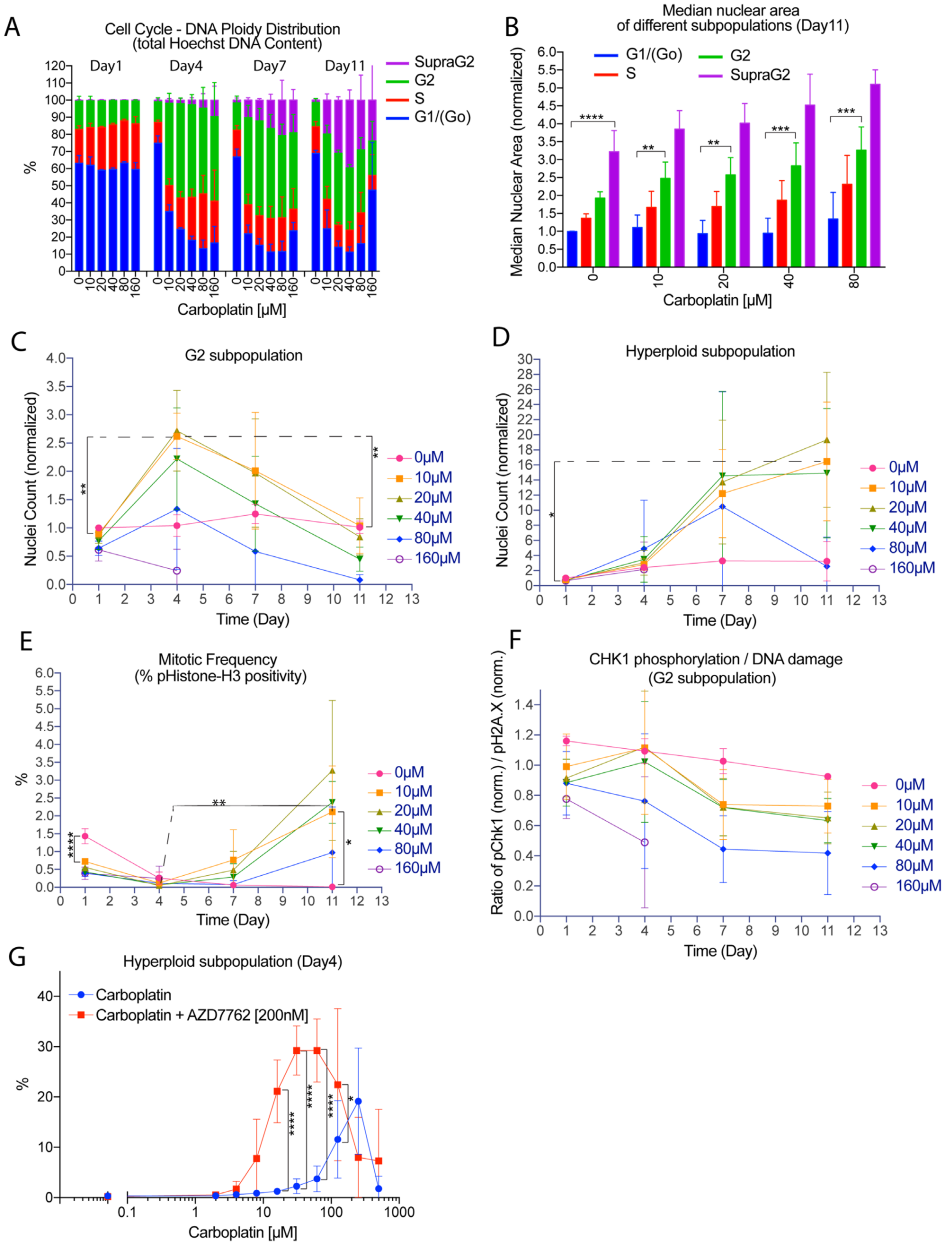
Nuclear area and DNA ploidy analysis revealed the *de novo* generation of a subpopulation of large hyperploid tumor cells due to G2-M checkpoint deregulation. Time course or dose-response experiment where adherent OVCAR3 cells maintained within 96-well plates were treated with carboplatin from 0 µM and up to 1000 µM for 24 hrs on day 0, followed by removal of the drug. For the time course experiment, one plate from a set of four was then fixed on day 1, 4, 7 or 11 for immunofluorescence or Hoechst staining. Only propidium-iodide excluded nuclei were used for quantification. (**A**) Time course of cell cycle phase/DNA ploidy distribution by total Hoechst DNA content. Data bars were the mean percentage of each subpopulation +/– standard deviations from 3 independent experiments. Nuclei analyzed ranged from 0 to about 20000 nuclei per well. (**B**) Median nuclear area of different subpopulations among the surviving cells on day 11 after carboplatin treatment. Data bars were the average of the median nuclear area of the indicated subpopulation (normalized to the Go/G1 of control) +/– standard deviations from 3 independent experiments. Median nuclear area assessed from well with at least 5 to about 20000 nuclei. Wells from the 160 µM carboplatin treatment were not included for analysis due to the very low number of surviving cells. ^**^ *p* < 0.01, ^***^ *p* < 0.001 and ^****^ *p* < 0.0001 using two-way ANOVA analysis with Bonferroni’s correction to demonstrate statistically significant differences between the G1/(Go) and the indicated subpopulation. (**C**–**D**) Time course showing nuclei count for the G2 subpopulation (C), or the hyperploid subpopulation with supra G2 DNA content (D) after carboplatin treatment with the indicated concentration. Data points were mean nuclei count per well (normalized to 0 µM of the G2 (C) or hyperploid (D) subpopulation on day 1) +/– standard deviations from 3 independent experiments. Nuclei analyzed ranged from 0 to about 20000 nuclei per well. ^*^ *p* < 0.05 and ^**^ *p* < 0.01 using two-way ANOVA analysis with Bonferroni’s correction to demonstrate statistically significant differences for the 10 µM treated condition between different day. (**E**) Time and concentration-dependent effect of carboplatin treatment on mitotic frequency as determined by percentage of nuclei positive for phospho-Histone-H3 staining. Positivity for pHistone-H3 staining was defined at the midpoint of the pH3 mean nuclear intensity histogram showing clear bimodal distribution. Data points were the mean percentage of pHistone-H3 positivity +/– standard deviations from 3 independent experiments. Percentage of pHistone-H3 positivity assessed from wells with at least 10 and up to about 20000 nuclei. ^*^ *p* < 0.05, ^**^ *p* < 0.01 and ^****^ *p* < 0.0001 using two-way ANOVA analysis with Bonferroni’s correction to demonstrate statistically significant differences between the different treatment conditions. (**F**) Time course showing the ratio of the normalized pCHK1 response to the normalized DNA damage burden (CHK1 pS345 / H2A.X pS139) for the G2 subpopulation. The ratio was also normalized to the bulk control population of each day. Data points were mean values +/– standard deviations from 3 independent experiments. Nuclei analyzed ranged from 0 to about 20000 nuclei per well. Wells with fewer than 5 cells were not included for analysis. (**G**) Percentage of the hyperploid subpopulation on day 4 after initial treatment on day 0 with carboplatin, followed by addition of vehicle or the CHK1 inhibitor AZD-7762 [200 nM] on day 1. Data points were mean percentage +/– standard deviations from 3 independent experiments. Nuclei analyzed ranged from 0 to about 24000 nuclei per well. ^*^ *p* < 0.05 and ^****^ *p* < 0.0001 using two-way ANOVA analysis with Bonferroni’s correction to demonstrate statistically significant differences between carboplatin treatment alone and the combination treatment.

We applied this analytical method to assess changes in the cell cycle – DNA ploidy status of tumor cells after platinum treatment. We observed the accumulation of G2-arrested OVCAR3 cells (6N) on day 4 after carboplatin treatment (Figure 3A), consistent with the known effect of this drug to induce G2 arrest [20]. Morphologically, the G2-arrested cells were moderately enlarged, with nuclear area 2 to 3-fold higher than cells in Go/G1 phase with 3N DNA content (Figure 3B). Interestingly, there was a hyperploid subpopulation with supra G2 Hoechst DNA content (>6N) that emerged on day 4 and its relative representation within the surviving population ranged from 15% to 40% on day 11 in a carboplatin concentration-dependent manner (Figure 3A). In addition, the hyperploid cells exhibited a 4-fold larger nuclear area when compared to cells with 3N DNA content (G1/Go) (Figure 3B). Therefore, we have identified the emergence of a subpopulation of large hyperploid tumor cells following carboplatin treatment.

To further assess the temporal relationship between the G2-arrested subpopulation and the hyperploid subpopulation, counting propidium-iodide-excluded, viable residual cells showed a biphasic kinetic pattern on the G2 subpopulation, with a maximum accumulation on day 4, followed by decline of this subpopulation (Figure 3C). This general trend was observed in all of the treatments except for the highest concentration of 160 µM where the G2 subpopulation exhibited steady decline. Following and mirroring the decline of the G2 subpopulation, there was a rise in the hyperploid subpopulation (not only in relative ratios but also in absolute numbers), although its major rise was delayed to day 7 for most of the treatments (Figure 3D). Therefore, we have quantitatively determined that platinum treatment induced a subpopulation of large hyperploid cells *de novo* and that this subpopulation arose as the G2-arrested subpopulation declined. This general pattern was modulated by treatment with the highest concentration of carboplatin (160 µM) in which both the G2 and the hyperploid subpopulation declined after treatment.

### Dynamic changes in the G2 and hyperploid subpopulation due to deregulation of the G2-M checkpoint

We inferred from the initial rise and subsequent fall of the G2-arrested subpopulation that the G2-M checkpoint might be deregulated at the intermediate to late time point (Day 7–11) critical for the generation of the hyperploid subpopulation. To address this issue, we correlated the kinetics of the emergence of the supra G2 subpopulation with the mitotic fraction and CHEK1 kinase (CHK1) activity to monitor a change in the status of the G2-M checkpoint.

The initial activation of the G2-M checkpoint after carboplatin treatment led to an accumulation of G2 arrested cells most evident by day 4 and a concordant inhibition of mitosis in this early time period. As assessed by the mitotic marker phospho-Histone-H3, the mitotic rate of OVCAR3 cells was reduced after 24 hours of carboplatin treatment in a concentration-dependent manner and was further inhibited by day 4 (Figure 3E). The G2-arrest and concomitant reduction of mitotic activity in carboplatin-treated cells may be attributed to the initiation of the DNA damage response by nuclear phospho-H2A.X staining (Supplementary Figure 4A–4H) and the subsequent activation of the CHEK1 kinase as assessed by serine 345 phosphorylation (Supplementary Figure 4I–4P). These cellular responses exhibited up to 2.6-fold increase in nuclear pH2A.X staining (Supplementary Figure 4D) and 2-fold increase in CHK1 serine 345 phosphorylation in G2-arrested OVCAR3 on day 1 (Supplementary Figure 4L). To further gauge the strength of the pCHK1 signal based on the amount of residual DNA damage burden, the pCHK1 response was normalized to the pH2A.X DNA damage response (Supplementary Figure 4Q–4U). The pCHK1/pH2A.X ratio also exhibited a biphasic kinetic pattern and its peak strongly correlated with the peak G2 arrest of carboplatin-treated cells on day 4 for all treatments except the highest concentration of 160 µM (Figure 3F). Therefore, the strength of CHK1 signaling from day 1 to day 4, and the accompanied G2-arrest and mitotic inhibition, all indicate a robust activation of the G2-M checkpoint.

To functionally demonstrate the role of CHK1 in maintaining G2-arrest, cells were treated with the CHK1 kinase inhibitor AZD-7762 between day 1 and day 4 after the initial carboplatin treatment. Figure 3G and Supplementary Figure 4V showed higher accumulation of hyperploid cells as early as on day 4 when checkpoint function was abolished, by percent representation or absolute count, respectively, indicating an earlier transition of G2-arrested cells to the hyperploid state.

Between day 4 and 7, the pCHK1/pH2A.X ratio declined substantially (Figure 3F). This change in pCHK1/pH2A.X ratio also corresponded to a fall in the G2 subpopulation (Figure 3C), followed by a new wave of mitotic activity as indicated by pHistone-H3 staining (Figure 3E). These results indicated an important reversal of the G2-M checkpoint status between the intermediate time point (day 4) and the later time points (day 7–11). Furthermore, the weakening of the G2-M checkpoint was tightly coupled to the rise of the supra G2 hyperploid subpopulation from day 7 to day 11, by absolute count (Figure 3D) or percent representation (Figure 3A).

For cells treated with the highest concentration of carboplatin (160 µM), the pCHK1/pH2A.X ratio exhibited a continuous declining pattern from the beginning, perhaps indicating an insufficiency of the pCHK1 response. This was also concordant with similar declining pattern of the respective G2 subpopulation as shown earlier (Figure 3C). These results suggested that high concentration of carboplatin treatment might impede the initial establishment of the G2-M checkpoint or expedite its deactivation.

### Time-lapse microscopy analysis revealed mitotic catastrophe as a cause of cell death and aberrant mitotic slippage events that may direct tumor cells toward a hyperploid state

**Figure 4:**
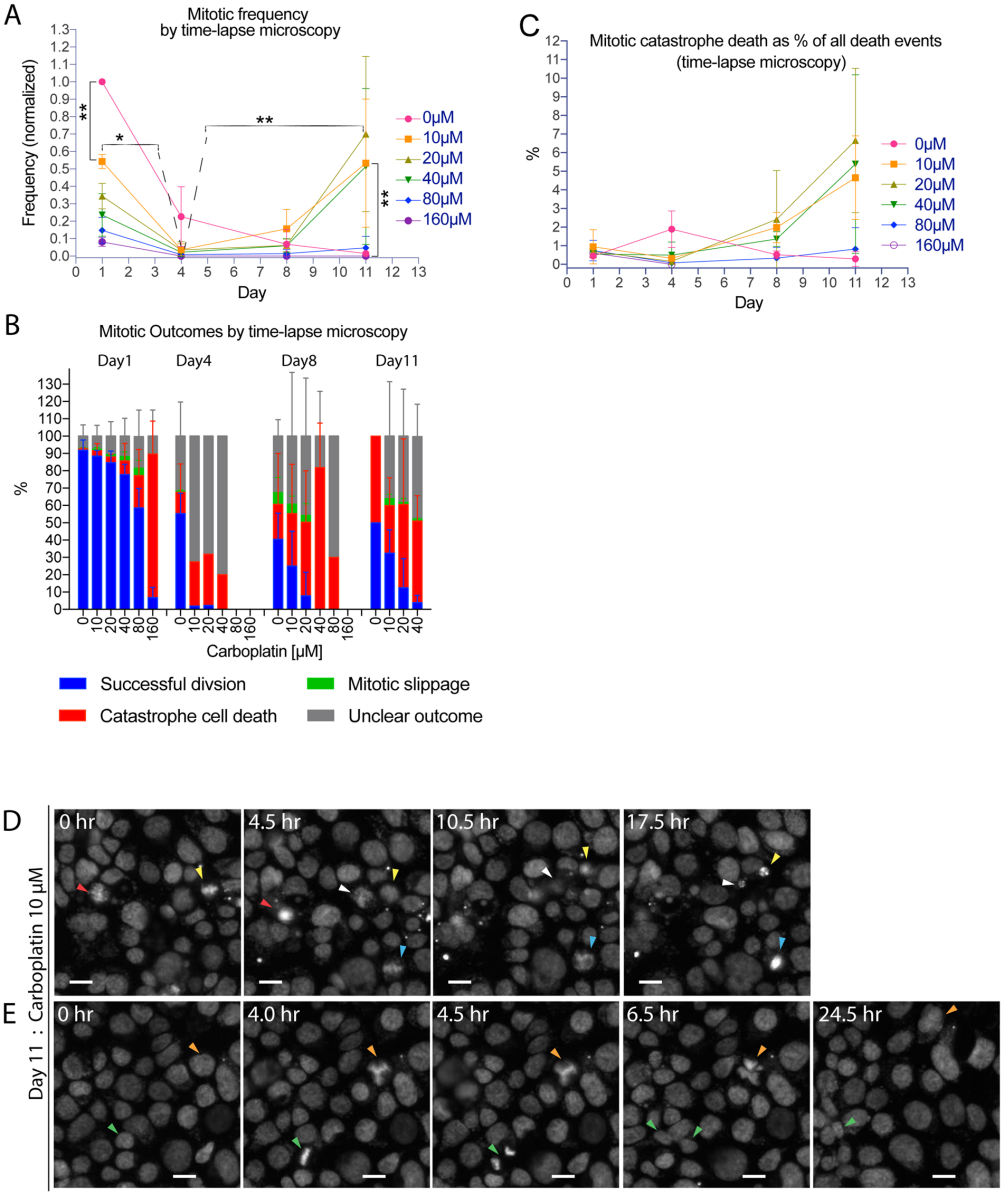
Time-lapse microscopy analysis revealed aberrant mitotic slippage events that may direct tumor cells toward a hyperploid state. Time-lapse microscopy experiment where OVCAR3 cells were treated with carboplatin (0 µM to 160 µM) on day 0 and then live-imaged on day 1, 4, 8 or 11 at half-hour interval for 10 to 24 hrs. For (**A**–**C**), nuclei count ranged from 0 to 10000 nuclei per well. Time-lapse recordings from 6 to 8 imaged fields within a representative well were visually analyzed for each treatment condition, from which there were 0 (i.e. 160 µM on day 11–12) to about 1000 mitotic events per well over a 10 to 24 hour-interval (i.e. control on day 1–2). A. Time and concentration-dependent effect of carboplatin treatment on mitotic frequency as determined by visual analysis of time-lapse recording of cells for mitotic events. Data points were mean frequency (normalized to the 0 µM condition on day 1) +/– standard deviation from 3 independent experiments. ^*^ *p* < 0.05 and ^**^ *p* < 0.01 using two-way ANOVA analysis with Bonferroni’s correction to demonstrate statistically significant differences between 0 µM and the indicated carboplatin concentration or between the same treated concentration on different day. (B) Percentage breakdown of mitotic outcomes from visual analysis of time-lapse recordings of live mitotic events: successful mitotic division with the generation of two or more viable daughter cells = blue, mitotic catastrophe cell death = red, mitotic slippage = green (defined as premature exit at metaphase-anaphase with no further progression to cytokinesis or re-unification of the daughter nuclei as one large irregularly lobated nucleus), unclear outcome = grey (which included mostly protracted mitoses stalled at metaphase or a smaller number of mitoses with ambiguous outcome due to technical issues from cells drifting out of focus/field or cell tracking). Data bars were mean percentage +/– standard deviations from 3 independent experiments. Conditions with fewer than 5 mitotic events were not shown for outcome analysis. (C) Contribution of mitotic catastrophe cell death to the overall cell death between day 1–2, 4–5, 8–9, or 11–12. Data points were mean mitotic catastrophe death as a percentage of all death events +/– standard deviations from 3 independent experiments. All death events ranged from 50 to 6000 events per well over a 10 to 24 hour-interval. (**D**) Images from time-lapse series of 10 µM carboplatin-treated cells undergoing mitotic catastrophe cell death on day 11 (arrowheads). Scale bar = 40 µm. (**E**) Images from time-lapse series of 10 µM carboplatin-treated cells going through mitotic slippage (orange arrowheads) or successful division (green arrowheads) on day 11. Scale bar = 40 µm.

The weakening of the G2-M checkpoint was tightly correlated with a new wave of mitotic activity and the rise of the hyperploid subpopulation, suggesting that checkpoint weakening could be coupled with mitotic progression and the emergence of hyperploidy. To ascertain whether there could be a mitotic outcome that may lead to a hyperploid state, we performed time-lapse recording of live Hoechst-stained OVCAR3 cells to follow the fate of mitotic events at various time points after carboplatin treatment. Time-lapse recordings showed a moderate level of mitotic activity for vehicle-treated OVCAR3 cells on day 1, but the mitotic frequency subsequently subsided between day 4 and day 11 (Figure 4A). More importantly, there was a significant carboplatin concentration-dependent reduction of mitotic activity between day 1 and day 4, followed by the emergence of a new wave of mitotic activity between day 8 and day 11 (Figure 4A). The demonstration of a biphasic pattern of mitotic activity by time-lapse microscopy complemented our earlier results using the pHistone-H3 mitotic marker (Figure 3E).

Several major types of mitotic outcomes were observed in the time-lapse recordings and were classified by the following categories: successful division, mitotic catastrophe cell death, mitotic slippage, or mitotic events with unclear outcome (see below). Whereas most vehicle-treated OVCAR3 cells on day 1 would undergo successful division leading to two daughter cells, most carboplatin-treated tumor cells that progressed through mitosis died as a result of mitotic catastrophe (Figure 4B, 4D). However, there were temporal and carboplatin concentration-dependent influences on the survival of these mitotically progressing tumor cells. Carboplatin treatment at 10 µM yielded a substantial percentage of cells that underwent successful mitosis (with a frequency of 25–30% on day 8–11), whereas carboplatin concentrations of 20–40 µM were permissive for viability on day 1 but rendered most cells dead at later time points (day 8, 11) (Figure 4B). Moreover, carboplatin treatment resulted in an increased number of mitotic events with unclear outcomes, which included mostly protracted mitoses stalled at metaphase with no further progression within the recording period and a much smaller number of mitoses whose outcomes could not be resolved due to technical reasons (i.e. loss from the visual field). Overall, the contribution of mitotic catastrophe-mediated death to the total number of cell death events induced by carboplatin was found to be 0.5%–6% (Figure 4C). However, the possibility should be considered that the mitotic death frequency could be somewhat underestimated due to technical reasons (i.e. recording frame rate, cell tracking difficulties). In any case, mitotic cell death was clearly detectable and might play a sizeable role in mediating the cytotoxic effects of platinum treatment.

In addition to cells that successfully completed mitosis or died by mitotic catastrophe, there was an alternate, non-lethal mitotic outcome through mitotic slippage events (Figure 4B). Mitotic slippage events were observed when cells initiated mitosis but prematurely exited this process by reverting back to the single-cell state, albeit with increased nuclear irregularities including extra lobation or multi-nucleation (Figure 4E, orange arrow head) [21]. Time-lapse recordings showed that higher concentration (40–80 µM) carboplatin treatments resulted in mitotic slippage events as early as day 1–2 whereas lower concentration treatments (10–20 µM) induced slippage events between day 8 and 12 (Figure 4B); however, technical factors associated with the time-lapse assay such as the recording frame rate (1 frame per 30 min) and method of cell tracking (visual/manual) could also potentially underestimate the mitotic slippage frequency. Based on the literature, it has been reported that a certain fraction of platinum induced damaged cells might undergo DNA endo-reduplication after mitotic slippage, resulting in hyperploid genome [13, 14, 22]. Our kinetic analysis of carboplatin treated cells showed temporal overlap between the mitotic slippage events (Figure 4B) and the emergence of the hyperploid subpopulation (Figure 3D), supporting the notion that mitotic slippage could serve as a mechanism to generate hyperploidy in HGSC cells treated with platinum based drugs [13, 14, 22]. Therefore, the deregulation of the G2-M checkpoint and the subsequent mitotic slippage process might direct tumor cells toward a hyperploid state.

### Size-sorted hyperploid subpopulation showed differential regrowth potential compared to the non-hyperploid fraction

Carboplatin treatment resulted in the generation of a large hyperploid subpopulation (>6N) as well as cells that remained in the baseline non-hyperploid state (3N-6N). To assess whether the baseline non-hyperploid (3N-6N) and the large hyperploid subpopulation (>6N) possessed differential clonogenic potential, these two subpopulations were enriched by flow sorting and evaluated for proliferation. Control or 25 µM carboplatin-treated OVCAR3 cells on day 9 were flow sorted by size (forward scatter) and plated at varying density on the 96-well plate. The size-sorting for control OVCAR3 cells provided some enrichment of small-size cells (Supplementary Figure 5A–5B and 5F) whereas size-sorting for carboplatin-treated cells yielded distinct small vs. large subpopulations (Supplementary Figure 5C–5E and 5F). There was a good correspondence between cell size as indicated by forward scatter (small or high FSC) and the median nuclear area, suggesting that size sorting was able to enrich for the large abnormal cells for evaluation (Supplementary Figure 5G). Importantly, the small-size post-sorted carboplatin-treated subpopulation corresponded to cells with predominantly baseline non-hyperploid Hoechst DNA content (3N-6N) (Supplementary Figure 5H: mean of 86% of sorted cells) whereas the large-size sorting provided a mean of 3.7-fold enrichment of the hyperploid subpopulation (>6N) (Supplementary Figure 5H: mean of 52% of sorted cells).

**Figure 5:**
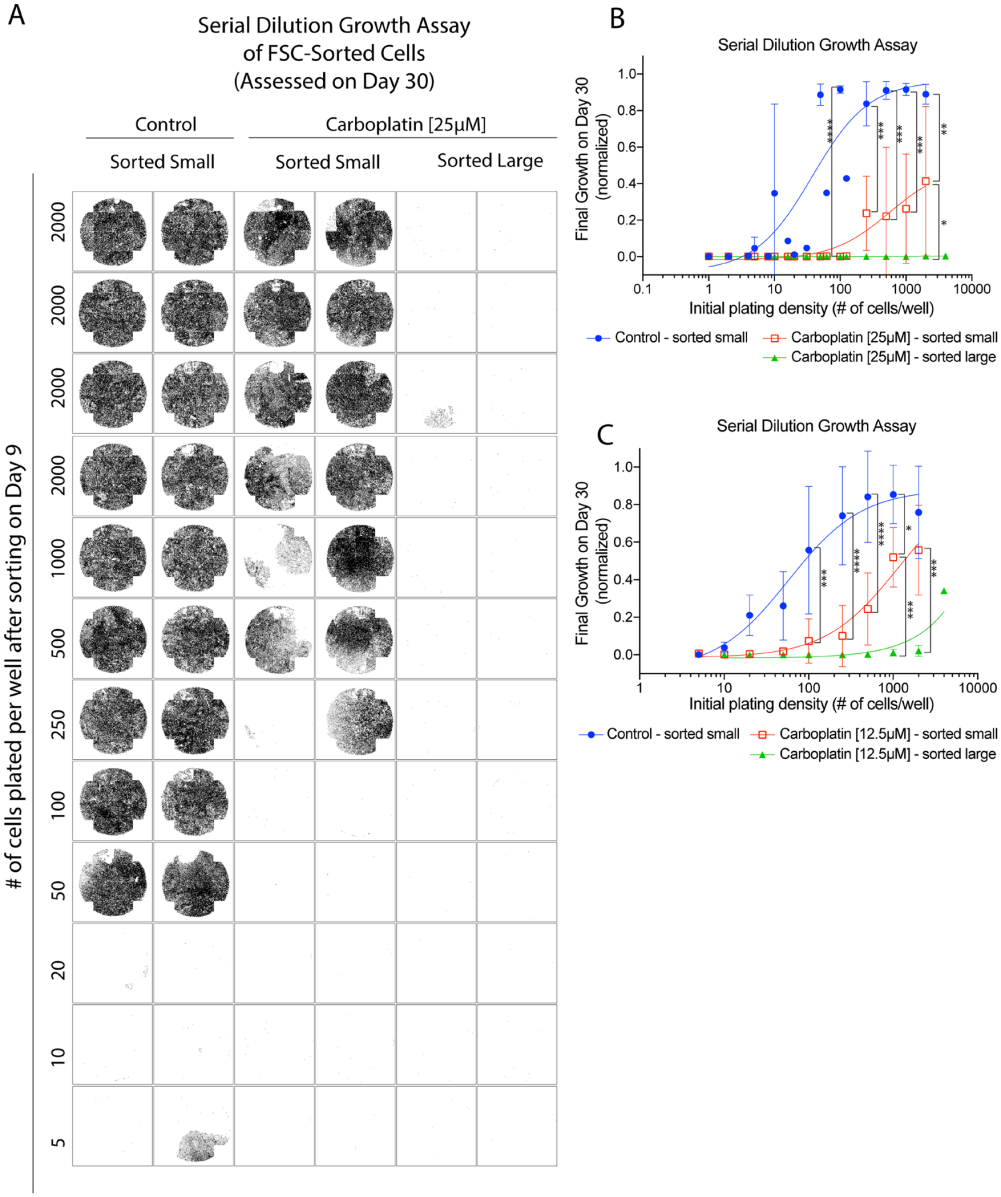
Size-sorted hyperploid subpopulation (>6N) showed differential regrowth potential compared to the non-hyperploid (3N-6N) fraction. Serial dilution growth assay where adherent OVCAR3 cells were treated with vehicle, 12.5 µM or 25 µM carboplatin on day 0 for 24 hrs, followed by removal of the drug and further incubation of the cells until day 9. Cells were then detached, flow sorted by forward scatter (FSC) and propidium-iodide negativity into a small or large subpopulation, and then plated into a 96-well plate at the indicated density for regrowth until final assessment by imaging on day 30. (**A**) The centroid location of each identified, propidium-iodide excluded, Hoechst stained cell within the wells of a 96-well plate was plotted on day 30, for the post-sorted control or post-sorted 25 µM carboplatin-treated subpopulation. (**B**) Final growth on day 30 as assessed by the total surface area of the well covered by tumor cell nuclei. The three groups were control (post-sorted FSC-small), or 25 µM carboplatin-treated (post-sorted FSC-small, post-sorted FSC-large). Data points were mean total covered surface area (normalized to the well with the largest growth) +/– standard deviations from 3 independent experiments. Nuclei analyzed ranged from 0 to about 27000 nuclei per well. (**C**) Final growth on day 30 from similar experiments as above where the three groups were control (post-sorted FSC-small), or 12.5 µM carboplatin-treated (post-sorted FSC-small, post-sorted FSC-large). Data points were mean total covered surface area (normalized to the well with the largest growth) +/– standard deviations from 3 sorting sessions. Nuclei analyzed ranged from 0 to about 67000 nuclei per well. ^*^ *p* < 0.05 and ^**^ *p* < 0.01, ^***^ *p* < 0.001 and ^****^ *p* < 0.0001 using two-way ANOVA analysis with Bonferroni’s correction to demonstrate statistically significant differences between the indicated post-sorted subpopulation.

The growth within each well of the 96-well plate on day 30 (since the original treatment) was assessed by imaging of each well in its entirety after viable cells were processed for staining. The centroid location of each propidium-iodide excluded, Hoechst-stained viable cell within each well was plotted and shown in Figure 5A. The growth potential of small-size post-sorted control OVCAR3 cells was demonstrated at or beyond an initial plating density of 50 cells per well. Carboplatin treatment reduced the growth potential of the small-size post-sorted subpopulation by about 5 to 10-fold (Figure 5A). For the large-size post-sorted subpopulation, there was more growth reduction, but not complete suppression as there was a rare area of growth in 1 of 8 wells plated at the 2000 cells per well density (Figure 5A). The growth potential of the large-size post-sorted carboplatin-treated subpopulation was estimated to be 1 in 20,000 cells, or about 40 to 80-fold less than the small-size post-sorted carboplatin-treated subpopulation. Results from three independent experiments were quantified in Figure 5B, where the growth within each well was assessed by percentage of surface area covered by the viable cell nuclei.

Because of the rarity of the growth events from the large-size post-sorted carboplatin-treated subpopulation, it was difficult to conclude whether these rare growth events were contributed by the hyperploid tumor cells or originated from the very small number of baseline non-hyperploid tumor cells that could not be separated from the large hyperploid subpopulation. Also, the viability of the tumor cells might be affected by the mechanical manipulation involved during the flow sorting process, thereby reducing the growth potential of tumor cells. We further repeated the growth assay using hyperploid cells derived from carboplatin treatment at a lower concentration (12.5 µM) (Supplementary Figure 5I–5J) and observed more outgrowths (Figure 5C). Overall, the findings from the growth assay performed after flow sorting indicated that the baseline non-hyperploid (3N-6N) carboplatin-treated subpopulation might predominantly contribute to the regrowth of the tumor cell line. Nonetheless, the occasional outgrowths from the post-sorted hyperploid subpopulation (>6N) raised the possibility of contribution by this subpopulation. This notion prompted us to further evaluate the viability status and growth potential of this population using other complementary approaches.

### Hyperploid tumor cells remained proliferative and retained capacity to generate viable daughter cells

**Figure 6:**
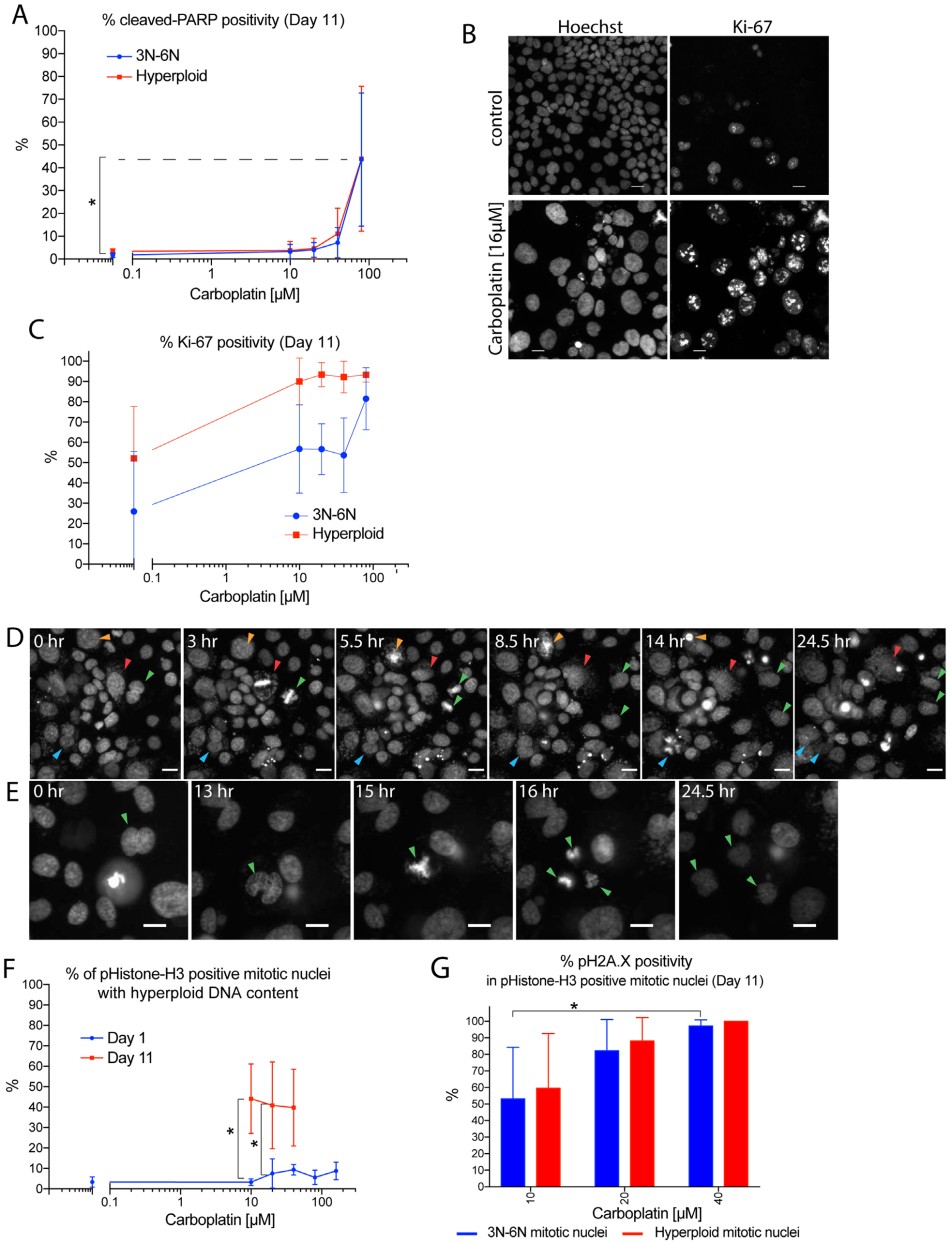
Hyperploid tumor cells remained proliferative and retained capacity to generate viable daughter cells. Immunofluorescence staining or time-lapse microscopy experiments where adherent OVCAR3 cells maintained within 96-well plates were treated with 0 µM to 160 µM carboplatin for 24 hrs on day 0, followed by removal of the drug. Residual cells within the wells were then fixed on day 11 for immunofluorescence and Hoechst staining (**A**–**C**, **F**–**G**), or imaged live by time-lapse microscopy between day 1–2, 4–5, 8–9, 11–12 (**D**–**E**). (A) Carboplatin concentration-dependent effect on percentage of non-hyperploid (3N-6N) or hyperploid cells with cleaved-PARP positivity on day 11. Cleaved-PARP positivity was defined using mean nuclear intensity and nuclear-to-cytoplasmic ratio of the antibody staining to identify cells (~2%) separated from the main viable cluster in the control bulk population. Data points were mean percentage of cleaved-PARP positivity +/– standard deviations from 3 independent experiments. Nuclei analyzed ranged from 20 to about 17000 nuclei per well. ^*^ *p* < 0.05 using two-way ANOVA analysis with Bonferroni’s correction to demonstrate statistically significant differences between 0 µM and the indicated carboplatin concentration. (B) Images showing control (top panels) or 16 µM carboplatin treated cells (bottom panels) on day 11 with Hoechst staining (left panels) or Ki-67 antibody staining (right panels). Scale bar = 20 µm. (C) Carboplatin concentration-dependent effect on nuclear Ki-67 antibody staining positivity for the non-hyperploid (3N-6N) or hyperploid subpopulation on day 11. Ki-67 positivity was defined as intensity above background. Data points were mean percentage of Ki-67-positivity +/– standard deviations from 3 independent experiments. Nuclei analyzed ranged from 10 to about 15000 nuclei per well. (D) Images from time-lapse series taken on day 11 (after prior carboplatin treatment of 10 µM) showing a mono-nucleated large cell (red arrowheads) and a bi-nucleated cell (blue arrowheads) going through mitotic slippage while a different bi-nucleated cell (green arrowheads) going through successful division. Another large mono-nucleated cell went through catastrophe cell death (orange arrowheads). Scale bar = 40 µm. (E) Images from time-lapse series taken on day 11 (after prior carboplatin treatment of 10 µM) showing a bi-nucleated cell going through successful tripolar mitosis and generating three daughter cells (green arrowheads). Scale bar = 40 µm. (F) Time and concentration-dependent effect of carboplatin treatment on percentage of phospho-Histone-H3 positive mitotic nuclei with hyperploid DNA content. Nuclei count ranged from 1 to about 15000 nuclei per well. Wells with fewer than five cells or five mitotic nuclei were not included for analysis. Data points were mean percentage of mitotic nuclei with hyperploid DNA content +/– standard deviations from 3 independent experiments. ^*^ *p* < 0.05 using two-way ANOVA analysis with Bonferroni’s correction to demonstrate statistically significant differences for the indicated carboplatin concentration between day 1 and day 11. (G) Concentration-dependent effect of carboplatin treatment on percentage of phospho-Histone-H3 positive mitotic nuclei with pH2A.X positivity on day 11. The mitotic nuclei were divided into those with non-hyperploid (3N-6N) vs. hyperploid DNA content for this analysis. pH2A.X positivity was defined in the control cells with staining intensity higher than the 99.5-percentile level. Nuclei count ranged from 1 to about 15000 nuclei per well. Wells with fewer than five cells or five mitotic nuclei were not included for analysis. Data bars were mean percentage of pH2A.X positivity +/– standard deviations from 3 independent experiments. ^*^ *p* < 0.05 using two-way ANOVA analysis with Bonferroni’s correction to demonstrate statistically significant differences for the non-hyperploid (3N-6N)-mitotic nuclei between the two indicated carboplatin concentration.

To determine whether the reduced growth potential from the large-size post-sorted hyperploid subpopulation was due to reduced viability of the large hyperploid cells, we first assessed the viability status of the large hyperploid cells after carboplatin treatment. Adherent OVCAR3 tumor cells within the 96-well plate on day 11 after carboplatin treatment were examined by antibody staining with an apoptotic marker, cleaved-PARP. The hyperploid cells were largely cleaved-PARP negative except for those derived from treatment with higher carboplatin concentration (80 µM) where cleaved-PARP positivity reached 50% (Figure 6A). To assess the proliferation status of the large hyperploid subpopulation, tumor cells were stained with an antibody to the proliferation marker Ki-67. The majority (80% to 90%) of the large hyperploid cells remained in the cell cycle whereas 20% to 50% of the baseline non-hyperploid subpopulation exited the cell cycle into quiescence (Figure 6B–6C).

As these results showed that the hyperploid tumor cells remained non-apoptotic and proliferative, we then assessed whether they may divide again to return to a non-hyperploid state by time-lapse microscopy. Live-cell recordings from OVCAR3 cells within the 96-well plate on day 11 after prior carboplatin treatment were analyzed. Importantly, there were large mono or bi-nucleated tumor cells with morphologic features characteristic of hyperploid cells that progressed through mitosis (Figure 6D–6E). While these unusual mitotic events may primarily lead to mitotic catastrophe cell death or slippage events (Figure 6D; red, blue, orange arrowheads), we observed that a small number of these mitotic events from the 10 µM carboplatin treatment resulted in successful completion of division, producing viable daughter cells (Figure 6D–6E; green arrowheads). Additional analysis of the mitotic nuclei at this late time point confirmed that the mitotic nuclei were larger in size (Supplementary Figure 6A) and 40–45% of them had supra G2 hyperploid Hoechst DNA content (Figure 6F), indicating that these unusual mitoses originated from hyperploid cells. Therefore, the viability status and the frequent mitotic progression of the hyperploid cells illustrated the transient nature of the hyperploid state, and signified a predilection toward the return to a non-hyperploid state.

There appeared to be lower extent of residual DNA damage, as shown by pH2A.X staining, associated with the hyperploid mitotic cells derived from the 10 µM carboplatin treatment, as compared to those generated from the higher carboplatin concentrations (20 µM – 40 µM) (Figure 6G, Supplementary Figure 6B–6D). This finding may explain the higher likelihood of successful mitotic completion by the hyperploid cells derived from the 10 µM carboplatin treatment. Therefore, the capacity to produce viable progenies through successful mitoses should prompt further consideration of the hyperploid pathway as a potential route to continue tumor cell survival.

## DISCUSSION

We have utilized an *in-vitro* experimental model system, coupled with a robust quantitative image-based analysis approach, to systematically study the cellular dynamics of tumor cell survivors after platinum treatment. This was motivated by observations from patient tumors of high grade serous carcinoma after neo-adjuvant chemotherapy treatment where a distinct surviving subpopulation of large bizarre tumor cells were observed in addition to the residual tumor cells retaining the usual morphologic features [15–17]. Subpopulation of large bizarre tumor cells has been reported in other cancer types after radiation or chemotherapy treatment, and may remain in the patient for many years. The biological potential and clinical implication of these large bizarre cells remain unclear.

We showed that platinum treatment induced cell cycle arrest at the G2-phase where the bulk tumor cell population may undergo DNA damage repair or initiate apoptotic death. While these findings were consistent with the established literature, we also demonstrated that subpopulations of cells exhibited nuclear enlargement as a result of exiting the G2-M checkpoint and eventually becoming hyperploid. Mechanistically, by assessing the strength of the regulator kinase CHK1, it was found that the level of phosphorylated CHK1 when normalized to the extent of DNA damage might be insufficient to maintain G2 arrest at intermediate to late time points. This led to aberrant mitotic progression where despite the overwhelming number of mitotic catastrophe cell deaths, there were occasional mitotic slippage events that provided a pathway toward cell survival through a subsequent genome polyploidization process.

Interestingly, similar mitotic slippage events have been described upon treatment of cells with microtubule-perturbing drugs [13, 14, 23–26]. These agents appeared to produce the initial activation of the spindle assembly checkpoint that stalled the progression of mitosis at the metaphase-anaphase transition but the durability of this blockade was time-limited, eventually giving rise to an escape route and leading to a tetraploid G1 state [27]. These escaped cells were observed to exhibit large nuclei with micronuclei formation and eventually developed polyploid genome [14, 28–30]. Indeed, we also observed the generation of similar hyperploid cells in the OVCAR3 *in-vitro* model after treatment with the microtubule polymerization inhibitor nocodazole (Supplementary Figure 7A–7H). Although these polyploid tumor cells have been shown to bear features of dormancy [14, 22, 25, 31], we showed by immunofluorescence staining that the hyperploid cells from our model system were Ki-67 positive and remained active in the cell cycle. However, this would not necessarily preclude the possibility of the hyperploid cells bearing markers of senescence and exhibiting certain senescence-like phenotypes, which might contribute, through secretory activities, to the survival of the bulk population, a possibility that warrants future studies. Importantly, additional colony regrowth assay demonstrated that while the non-hyperploid subpopulation may contribute to the majority of the regrowth, there was rare growth by progenies from the sorted hyperploid subpopulation. This result suggested that a small subset of the hyperploid cells might retain the capacity to repopulate among the regenerating tumor cell line. However, there were significant technical limitations inherent to the colony regrowth assay which might confound the interpretation of these results (i.e. possible unique sensitivity of the hyperploid subpopulation toward trypsinization, flow sorting process).

To overcome these limitations, we performed live-cell imaging analysis to follow the fate of the hyperploid subpopulation and showed that some of the large mono or bi-nucleated tumor cells could undergo successful division to generate viable daughter cells. Hence, the platinum-induced hyperploid state might not necessarily lead to dormancy. Rather, it might serve as an intermediate transition point for temporary reprieve of the ongoing stresses until cells are poised to revert back to a non-hyperploid state [32]. Because the capacity to produce viable daughter cells was demonstrated for the hyperploid cells, we believe the apparent low regrowth potential as ascertained from the earlier sorting and regrowth experiment does not definitively preclude the possibility that the occasional hyperploid cell could contribute to the regeneration of the tumor cell line. Future examination, utilizing more stringent single-cell tracking technique, will be useful to quantify the regrowth potential of the hyperploid subpopulation.

The generation of hyperploid cells was also observed in the OVCAR3 model after treatment with the topoisomerase II inhibitor and DNA intercalator doxorubicin, suggesting DNA damage as a common cause leading to this hyperploid response (Supplementary Figure 7I–7L). In addition, the hyperploid response was not specific to the OVCAR3 model as it was observed in the surviving population across several other ovarian cancer cell lines (TOV3133G, PEA1), indicating that this could be a generalized cellular response to platinum treatment (Supplementary Figure 7M–7O). The features of the tumor cell fraction within each cell line that entered this hyperploid pathway were not known and might largely be stochastic. We propose that a dysfunctional p53 pathway might be a contributor to occurrence of this state, as the various ovarian cancer cell lines and the SV40 large T-antigen transformed lung epithelial cell line (16HBE) that exhibited the hyperploid response all shared a commonality in p53 alteration as opposed to the non-transformed gingival fibroblast cell line (HGF-1) (Supplementary Figure 7P–7Q). Potentially, baseline perturbations of the G2-M cell cycle checkpoint due to p53 pathway alteration could lower the threshold governing the entry into the mitotic-hyperploid pathway after platinum treatment.

In general, the hyperploid pathway was not utilized until cell survival fell below 50% (Supplementary Figure 7O), suggesting that other anti-death processes such as anti-apoptotic signaling could be important for maintaining survival when the bulk population was treated with carboplatin at sub-lethal concentrations. When, however, the carboplatin-induced toxicity became overwhelming (i.e. bulk population was reduced to 10–20%), there was a substantial shift toward the hyperploid response (Supplementary Figure 7O). We further derived several matched platinum-resistant cell lines from the OVCAR3 parent (Supplementary Figure 7M), and demonstrated substantially lower hyperploid response in these daughter cell lines (5–30% for RES1; 2–10% for RES2) as compared to the parental cell line (10–40%), suggesting that a lower extent of hyperploid response after platinum treatment might be correlated with platinum resistance (Supplementary Figure 7R–7S). The lower utilization of the hyperploid pathway could be due to strengthening of the G2-M checkpoint by the platinum-resistant daughter cell lines, perhaps as an adaptation to curtail entry into the more perilous hyperploid pathway (Supplementary Figure 7T–7V).

**Figure 7:**
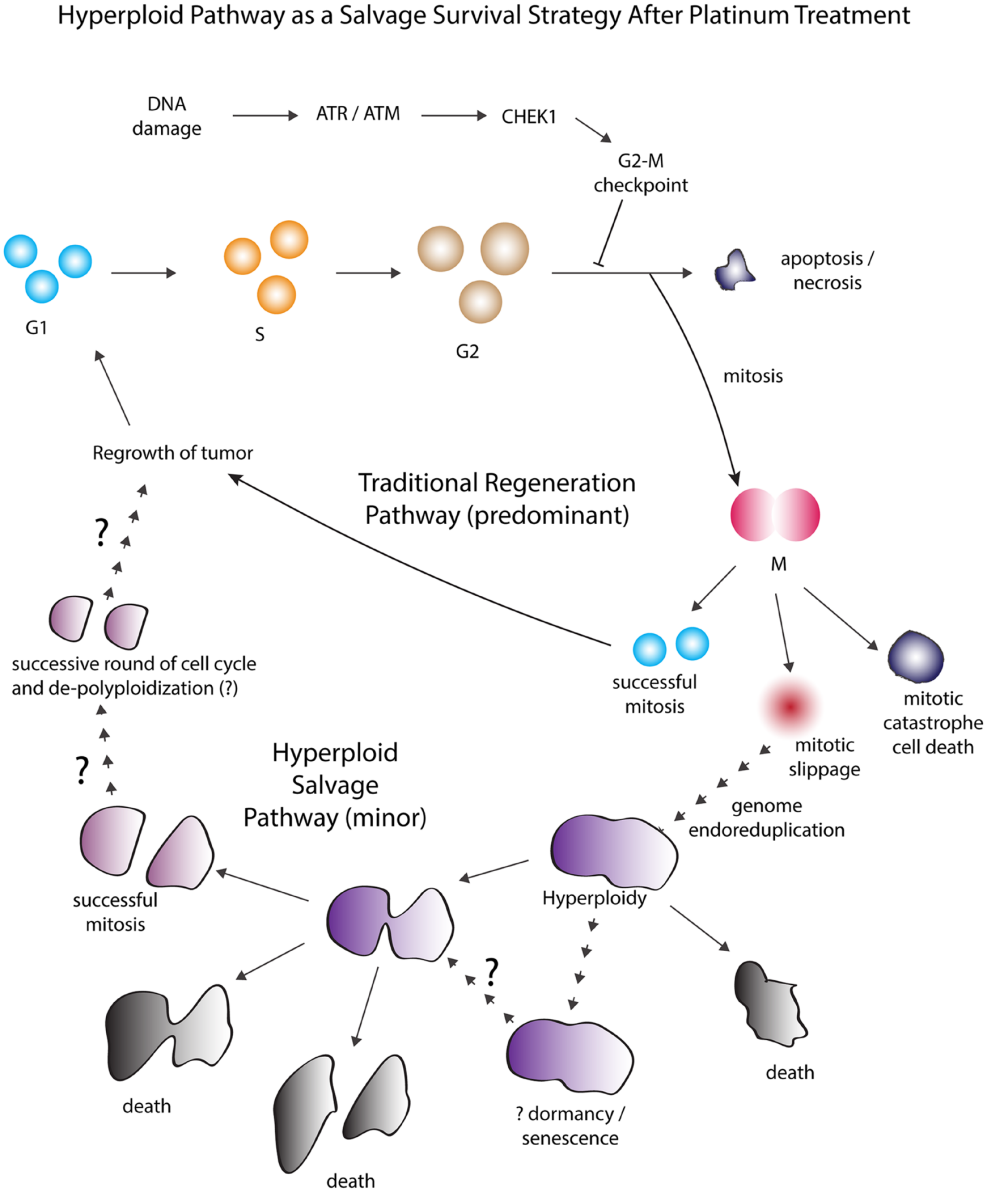
Proposed model of the hyperploid pathway as a salvage survival strategy regulated by the G2-M checkpoint. Hyperploid pathway as a salvage survival strategy after moderate to high but not extreme concentration of platinum treatment (i.e. 20–100 uM). After platinum treatment, the activation of the G2-M cell cycle checkpoint led to the initial arrest of tumor cells at the G2-phase for DNA damage repair. Under the traditional regeneration pathway, tumor cells that did not perform sufficient DNA damage repair would undergo cell death such as by apoptosis or necrosis whereas others that achieved higher level of repair would eventually emerge from the G2 arrest and progress through mitosis to produce viable daughter cells. These normal progenies would predominantly contribute to the regeneration of the tumor cell line. However, an alternate salvage survival pathway was observed in which some of the initially G2-arrested tumor cells with residual DNA damage burden could avoid apoptotic cell death and aberrantly progress through mitosis at an intermediate to late time point. The perilous entry into mitosis while carrying residual DNA damage led mostly to mitotic catastrophe cell death. But there were occasional tumor cells that managed to exit mitosis via mitotic slippage and thereby avoided mitotic death. Following mitotic slippage, subsequent less-defined processes thought to result in the duplication of the genome led these cells to a hyperploid state. Although these *de novo* generated hyperploid tumor cells may continue to persist in this state, some may die while a substantial fraction would undergo mitosis. The chance for successful mitotic completion depended critically on the initial platinum treatment concentration and only those that were derived from the lowest treatment concentration could produce viable progenies. It remains to be further explored whether and how these unique progenies would contribute to the diversity of the regenerating tumor cell line.

We propose an overall model (Figure 7) wherein bypassing of apoptosis and entering into a mitotic-hyperploid pathway after carboplatin treatment might represent a salvage survival strategy, a state that occurs in the tumor cell population when challenged with a moderate to high level (i.e. 20 uM–100 uM) of carboplatin. The efficacy of this pathway to produce viable progenies was low and highly contingent on the initial platinum concentration that generated the hyperploid cells. The high attrition rate due to cell death throughout the different stages of this pathway would largely reduce the initial G2-arrested fraction to the few survivors that might emerge after the hyperploid cycle. Our study provided evidence that these rare progenies might reside within the regenerating tumor cell line. Potentially, the coupled process of hyperploidization and de-polyploidization might increase the genomic diversity of the recovering tumor cell line and contribute to acquired platinum resistance. Future studies are warranted to define the clinical significance of these hyperploid cells by correlating their frequency with clinical outcomes (i.e. overall and progression-free survival).

## MATERIALS AND METHODS

### Cell culture

Cell lines used in this study have been previously described: OVCAR3 [19], TOV3133G [33], PEA1 [34], 16HBE [35], HGF-1 (ATCC CRL-2014) [36]. The OVCAR3, TOV3133G and PEA1 ovarian cancer cell lines were maintained in RPMI 1640 (Wisent Bioproducts 350-000-CL) and OSE media (Wisent Bioproducts 316-030-CL) at a 1:1 ratio and with 6% heat-inactivated fetal bovine serum (Invitrogen 12484028) and 1% penicillin-streptomycin (Gibco 15140122). The 16HBE SV40 large T antigen transformed lung epithelial cell line and the HGF-1 human gingival fibroblast cell line were maintained in DMEM (Wisent Bioproducts 319-005-CL) with 6% FBS and 1% penicillin-streptomycin. Cells were plated onto Corning 96-well plates (half area, model #3882, VWR #33500-996) for experiments.

### *In-vitro* generation of OVCAR3 carboplatin-resistant daughter cell lines

The OVCAR3 carboplatin-resistant cell line RES_1 was generated by treating the parental OVCAR3 cell line with 10 µM carboplatin for 24 hours each time, for a total of 6 treatments, with approximately one-month interval between each treatment. The OVCAR3 carboplatin-resistant cell line RES_2 was generated by treating the parental OVCAR3 cell line with 90 µM carboplatin for 24 hours, followed by a two month interval, before a second treatment with 100 µM carboplatin for 24 hours, and followed by a one-month interval before a third treatment of 100 µM carboplatin for 24 hours.

### Cell viability staining and assessment

Cells were live-stained with 5 µM propidium iodide (Sigma Aldrich P4170) and / or 10 µL/mL of CellEvent Caspase 3/7 Green indicator (Molecular Probes R37111) for 30 min at 37C, for assessment of viability status before downstream live imaging or PFA fixation. For routine evaluation of cell survival of each well within the 96-well plate, cells were processed for PFA fixation and downstream permeabilization and Hoechst 33342 staining (2 uM) for 30 min, followed by PBS washing. 12 or 15 fields within each well of the 96-well plate were imaged by the INCELL 6000 platform, followed by automated nuclei detection and mean nuclear fluorescence quantification (see below). Single cell level data were prepared (see below) and then analyzed within the FlowJo program (see below). A minimum threshold level was set for the integrated Hoechst nuclear intensity (nuclear area x mean Hoechst nuclear fluorescence intensity) to help identify nuclei and exclude small / disintegrated DNA debris. Then, a threshold level was set for the mean nuclear propidium iodide fluorescence intensity, above which nuclei were defined as propidium-iodide-positive and interpreted as non-viable. The propidium-iodide negative and Hoechst-positive nuclei count was taken as the viable cell count within the well. In a typical experiment with cytotoxic treatment (i.e. carboplatin), three technical replicates (wells) were analyzed for each condition and the mean viable cell counts for each condition were calculated. The mean viable cell counts for a typical time-course experiment (day 1, 4, 7, 11) were normalized by the mean viable cell count from the vehicle-treated wells on day 1, thereby setting cell survival as 1 for the control well on day 1.

### Pharmacological treatments

Carboplatin (S1532), doxorubicin (S1208), the pan-caspase inhibitor Emricasan (S7775), and the CHK1 kinase inhibitor AZD-7762 (S1532) were obtained from Selleckchem. Carboplatin or doxorubicin treatment was applied to cells on day 0 for 24 hours and then removed on day 1. Where indicated, cells were incubated with Emricasan at 2 µM or 5 µM from day 0 or day 1 until the end of the experiment. Emricasan was refreshed every three to four days with change of media and fresh drug incubation. AZD-7762 was added on day 1 after carboplatin was removed, and then maintained throughout the rest of the experiment. AZD-7762 was refreshed every three to four days with change of media and fresh drug incubation. Nocodazole (M1404) was obtained from Sigma-Aldrich and applied to cells at 50 nM or 200 nM on day 0 continuously for 3 days, followed by fixation and downstream processing for staining and analysis.

### Time course experiments

Cells were plated onto a set of four 96-well plates at 8,000 cells/well three days before carboplatin treatment (day -3), and would usually grow to 95–100% confluence over the three days. On day 0, carboplatin treatment was performed with a concentration scheme of 0 µM, 10 µM, 20 µM, 40 µM, 80 µM, or 160 µM for 24 hrs. On day 1, carboplatin was removed and cells were incubated with fresh media. One plate was fixed with PFA 4% for 30 min on day 1, 4, 7 or 10–12. The last time point on day 10–12 would be listed as day 11 throughout the manuscript. Media was exchanged every three to four days for the remaining plates of the experiment.

### Cell line validation

STR-GenePrint analysis of the OVCAR3 parental and the matched *in-vitro* derived resistant daughter cell lines (RES_1 and RES_2) were performed by the Sickkids Centre for Applied Genomics and Genetic Analysis Facility and matched the published profile. The STR Geneprint analysis was also performed for the TOV3133G cell line.

### Immunofluorescence protocol

Adherent OVCAR3 tumor cells within 96-well plates were fixed with 4% paraformaldehyde (Electron Microscopy Science 15710) for 30 min and then permeabilized with 0.15% Triton X-100 (Sigma-Aldrich X100) overnight at 4C. Blocking was performed with 5% goat serum (Gibco 16210072) for 1 hour before primary antibody incubation overnight at 4C and then incubation with fluorescently-conjugated secondary antibody at 1:2000 for 3–5 hrs. Nuclear staining was performed with Hoechst 33342 (Molecular Probes H1399) at 2 µM for 30 min. At least three to four separate PBS washes were then performed before imaging.

Primary antibody incubation buffer (40 µL/well): 0.015% Triton X-100, 0.1% bovine serum albumin (ALB001.250). Secondary antibody incubation buffer (40 µL/well): 0.1% bovine serum album (ALB001.250).

#### Primary antibodies

Ki-67 (8D5) (CST9449, mouse, 1:500); Cleaved-PARP (CST5625, rabbit, 1:350); H2A.X phospho-serine 319 (clone JBW301) (EMD 05-636, mouse, 1:800); Histone-H3 phospho-serine 10 (D2C8) (CST3377, rabbit, 1:600); CHEK1 phospho-serine 345 (CST2348, rabbit, 1:200).

#### Secondary antibodies

Goat anti-mouse IgG (H+L) Superclonal secondary antibody, Alexa Fluor 488 conjugate (Molecular Probes A28175); Goat anti-mouse IgG (H+L) F(ab’)2 fragment secondary antibody, Alexa Fluor 647 conjugate (CST4410); Goat anti-rabbit IgG (H+L) Superclonal secondary antibody, Alexa Fluor 555 conjugate (Molecular Probes A27039); Goat anti-rabbit IgG (H+L) Superclonal secondary antibody, Alexa Fluor 647 conjugate (Molecular Probes A27040); Goat anti-rabbit IgG (H+L) highly cross-adsorbed secondary antibody, Alexa Fluor 488 conjugate (Molecular Probes A11034).

### Pulse labeling with EdU and Click-iT reaction [37]

Live cells were incubated with 10 µM EdU (5-ethynyl-2’-deoxyuridine) (Molecular Probes, C10340) for 30 min at 37C to label the S-phase fraction. Cells were then fixed with 4% PFA and permeabilized with Triton X-100% at 0.15%.

The Click-iT reaction was performed by sequential addition of copper sulfate pentahydrate (CuSO_4_-5H_2_O) [1 mM final] (BioShop CUS803.250), sulfo-cyanine5 azide [10 µM final] (Lumiprobe B3330) and sodium ascorbate [100 mM final] (BioShop ASO704.100) into a reservoir of Tris buffer [100 mM pH8.5] (BioShop TRS001.5). The sodium ascorbate solution was prepared fresh from powder each time. Each component was added into the reservoir and mixed well. 40 µL volume of the final reaction mixture was then pipetted into each well for incubation time of 15 min. Cells were then incubated with a PBS wash containing 1.5% BSA for 15 min.

### High-throughput time-lapse imaging of live cells

For preparation of each live-imaging session, OVCAR3 cells were plated onto different sections of the same 96-well plate on four separate occasions. Cells were then allowed to grow to confluence over three days before being treated with carboplatin (0 µM to 160 µM) at 1, 4, 8, or 11 days before imaging. Each treatment condition was technically repeated with four wells within the plate. Carboplatin was removed 24 hours after the onset of treatment. Immediately prior to imaging, cells were first incubated with Hoechst 33342 at 0.8–1 µM for 30 min at 37C before removal of the Hoechst dye. Then, cells were kept in media containing 5 µM propidium iodide and 10 µL/mL of the CellEvent caspase 3/7 green indicator. Hardware (plate-based) auto-focusing followed by imaging was performed every half-hour for a total of 10 to 24 hours on INCELL Analyzer 6000 (GE Healthcare Life Sciences) at 37C and with supply of humidified CO_2_. Images from six to eight random fields per well were acquired.

### High-throughput imaging of fixed cells

96-well plates were imaged using the INCELL Analyzer 6000 high-content laser scanning confocal microscopy system. A set of twelve randomly placed fields within each well were imaged, representing up to 30,000 cells/well. Up to four channels (Hoechst/DAPI, FITC, RED, CY5) were acquired for each field. Imaging was performed with hardware (plate-based) auto-focusing.

Objective: Nikon 20X (NA = 0.45 Plan Fluor ELWD with working distance of 7–8.1 mm; NA = 0.75 Plan Apo with working distance of 1 mm); sCMOS Camera (2048 x 2048 pixels at 16-bit depth); Excitation with four laser lines: 405 nm, 488 nm, 561 mm, 640 nm; Emission was captured with polychroic emission filter: 420–490 nm (Hoechst/DAPI), 505–545 nm (FITC), 569–641 nm (RED), 660–753 nm (CY5).

### Image processing and analysis

Automated image segmentation was performed using a custom image analysis routine for Acapella 2.6 (PerkinElmer). Image analysis script is available upon request. Cell-level data was saved in csv files for each well and then converted into FCS format using the freeware TextToFCS^*^. FCS files were imported into FlowJo (Flowjo, LLC) cytometry software for analysis.

^*^ http://flowjo.typepad.com/the_daily_dongle/2010/12/roll-your-own-fcs-files-part-2.html.

For each FCS file, the integrated Hoechst nuclear intensity (i.e. Hoechst DNA ploidy histogram) was derived by the multiplication of the nuclear area with the mean Hoechst nuclear intensity. Gating was applied on the integrated Hoechst nuclear intensity to separate the subG1 region (debris) from the rest. The propidium iodide nuclear intensity was then used to identify propidium-iodide excluded cells for viability cell count analysis. Cell cycle gating was then applied onto the Hoechst DNA ploidy histogram, with the position of the G1/(Go), S, G2 gates obtained from the EdU horseshoe plots (EdU mean nuclear intensity vs. integrated Hoechst nuclear intensity). An additional supraG2 gate was applied to the right end of the G2 gated region. Numerical data was then exported into Prism 7 (GraphPad Software) for plotting and statistical analysis.

### Time-lapse recording analysis

Mitotic events were visually analyzed and categorized as successful division with the generation of two or more daughter cells, mitotic catastrophe cell death, mitotic slippage, or unclear outcome. All death events were calculated as followed: nuclei count (at beginning of recording) + # of successful mitoses over the duration of recording – nuclei (at end of recording). The rate of cell death per hour was the cell death event per hour as a percentage of the starting nuclei count. Six to eight imaged fields within one representative well were quantified for each treatment condition, representing from 0 (i.e. 160 µM on day 11–12) to 10000 cells (i.e. control on day 1–2), and from 0 (i.e. 160 µM on day 11–12) to 1000 mitotic events (i.e. control on day 1–2).

### Flow sorting

OVCAR3 cells were either plated within T25 flask and treated with vehicle as control, or plated within T125 flasks and treated with 12.5 µM or 25 µM carboplatin for 24 hour. Carboplatin was removed on day 1. On day 9 after treatment, cells were detached by trypsinization, followed by trypsin inactivation with cold PBS supplemented with 5% FBS, 1% penicillin-streptomycin, 0.1 mM EDTA (BioShop EDT001.500). Cells were then spun down and incubated on ice in PBS supplemented with 0.5% FBS, 1% penicillin-streptomycin, 0.1 mM EDTA, 5 mM HEPES, 1 µM propidium iodide, and 5 µM Emricasan. Cells were then passed through 100 µm nylon mesh cell strainer (Falcon 352360). Between 2 to 5 million cells were harvested for each experiment, and 1 million cells were then pipetted into each polypropylene tube for sorting.

For the sorting experiments with 25 µM carboplatin-treated cells, three independent experiments involving three separate sorting sessions were performed. For the sorting experiment with 12.5 µM carboplatin-treated cells, two independent experiments were performed. The first experiment was performed with a sorting session on day 9. The second experiment included two different sorting sessions on day 8 and day 12 (using additionally prepared flasks).

Sorting was performed by dedicated technical staffs at the Hospital for Sick Children flow cytometry facility. The MoFlo Astrios Sorter (Beckman Coulter Life Sciences) was used for cell sorting. Sorting was performed by forward scatter (FSC) on a linear scale in which FSC distribution ranged from 0 to 256. For the control OVCAR3 sample, a FSC gate was placed from 10 to 64 to define a ‘control – small’ gate for sorting. For the OVCAR3 carboplatin treated samples, the same FSC gate from 10 to 64 was placed to define a ‘Carboplatin – small’ gate while another gate from 150 to 210 was placed to define a ‘Carboplatin – large’ gate for sorting. Sorted cells were directed by the flow sorter either into each well of a 96-well plate or into polypropylene tubes.

### Serial dilution regrowth assay

Sorted cells were placed into each well of a 96-well plate at different plating density of 5 cells/well to 2000–4000 cells/well. Cells were also incubated with the pan-caspase inhibitor Emricasan (5 µM) for the entire duration of the regrowth assay. Emricasan was refreshed every three to four days with change of media and fresh drug incubation. The readout of cell growth was performed at three weeks after the flow sorting, which corresponded to around day 30 (30 days after the initial carboplatin treatment). Cells were incubated with propidium iodide before fixation and permeabilization for Hoechst staining. The plate was then imaged where every well was scanned in its entirety using the INCELL Analyzer 6000 platform. The total nuclear count was quantified. The pattern of growth was also examined by plotting the x and y coordinate of each propidium-iodide excluded cell within the well in FlowJo. This enabled the identification of growing colonies as well as sparsely adherent single cells within the well. The total surface area of the well covered by the tumor cell nuclei was also quantified and was a good approximation of the extent of colony growth.

### Pathology review of patient high grade serous carcinoma

Patient cases of high grade serous carcinoma from the Laboratory Medicine Program at the University Health Network (UHN), Toronto were reviewed. Archived H&E slides of ten cases of post neo-adjuvant chemotherapy treated HGSC and five cases of treatment-naïve HGSC were reviewed. Research ethics approval was obtained and research conduct guidelines were followed.

## SUPPLEMENTARY MATERIALS


