# Supplementary material for: Avoidance of apoptotic death via a hyperploid salvage survival pathway after platinum treatment in high grade serous carcinoma cell line models
Source: Oncotarget. 2019 Nov 19;10(62):6691–712. doi: 10.18632/oncotarget.27330 (PMC6877103; doi:10.18632/oncotarget.27330)
Supplement: Supplementary file 1 [file oncotarget-10-6691-s001.pdf]

Avoidance of apoptotic death via a hyperploid salvage survival pathway after platinum treatment in high grade serous carcinoma cell line models

SUPPLEMENTARY MATERIALS

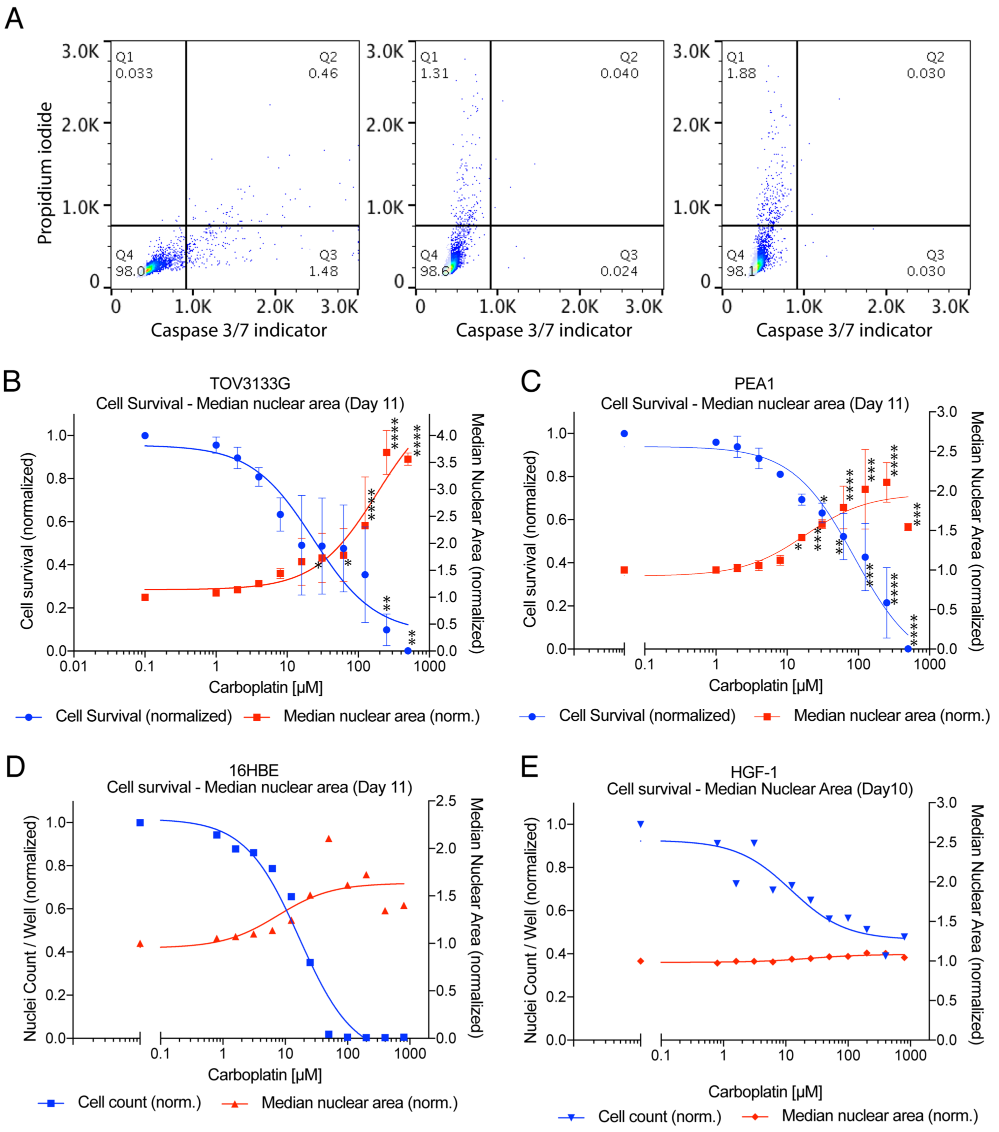

Supplementary Figure 1: Carboplatin concentration-dependent effect on cell survival and median nuclear area. (**A**) Adherent OVCAR3 cells were treated with vehicle (left panel; 12304 nuclei) or Emricasan at 0.5 uM (middle panel; 12481 nuclei) or 1 uM (right panel; 13441 nuclei) from day 1 to 7 and then live-stained with the Caspase 3/7 indicator and propidium iodide on day 10 before imaging. Data presented was from one of two representative experiments, and also representative of the TOV3133G cell line. (**B–E**) Adherent cells from the different cell lines were treated with the indicated carboplatin concentration for 24 hrs on day 0, followed by drug removal. Carboplatin concentration-dependent effect on cell survival (blue curve) or median nuclear area (red curve) on day 11 was shown for the TOV3133G (B), PEA1 (C), 16HBE (D) or HGF-1 (E) cell line. Data points for B and C were mean cell survival (propidium-iodide excluded nuclei count per well; normalized to 0 µM condition) or average of median nuclear area (normalized to 0 µM condition) +/- standard deviations from 3 independent experiments. For D and E, data points were mean from technical replicates of one experiment. Nuclei analyzed ranged from 15 to about 27000 nuclei per well for B, from 0 to about 6000 nuclei per well for C, from 30 to about 27000 nuclei per well for D, and from 460 to about 4000 nuclei per well for E. $^{*}p < 0.05$, $^{**}p < 0.01$, $^{***}p < 0.001$, and $^{****}p < 0.0001$ using two-way ANOVA analysis with Bonferroni's correction to demonstrate statistically significant differences between 0 µM and the indicated carboplatin concentration.

High grade serous carcinoma - Case #1 (post neo-adjuvant chemotherapy)

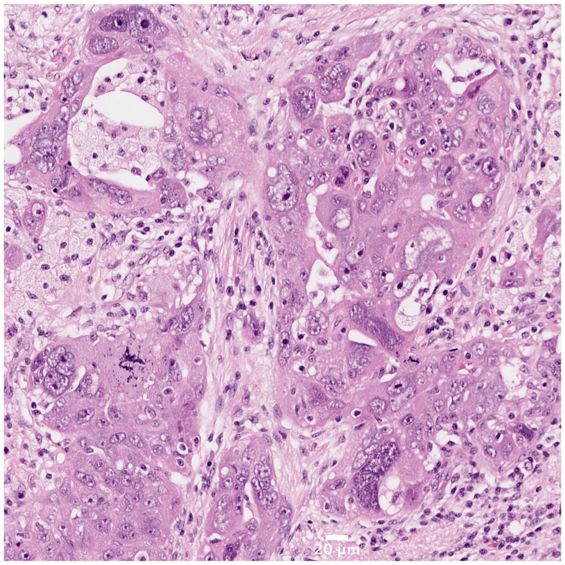

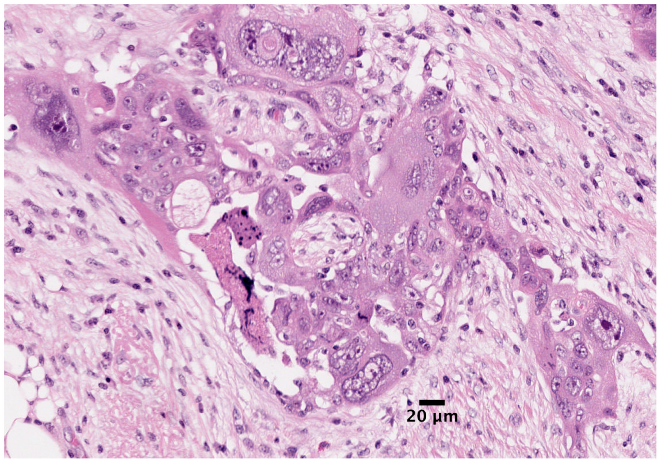

High grade serous carcinoma - Case #2 (post neo-adjuvant chemotherapy)

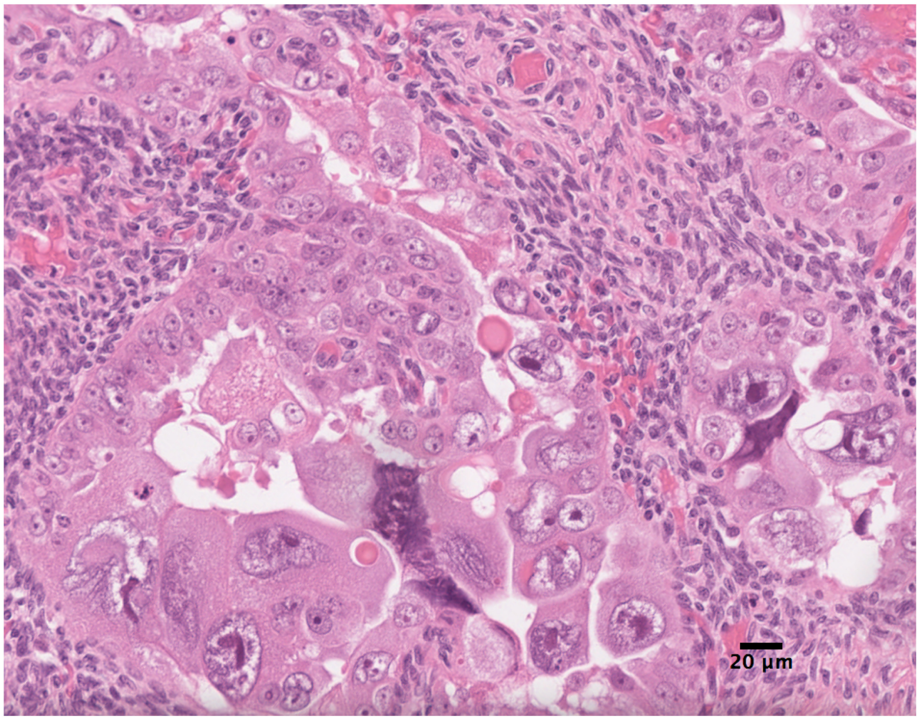

High grade serous carcinoma - Case #3 (post neo-adjuvant chemotherapy)

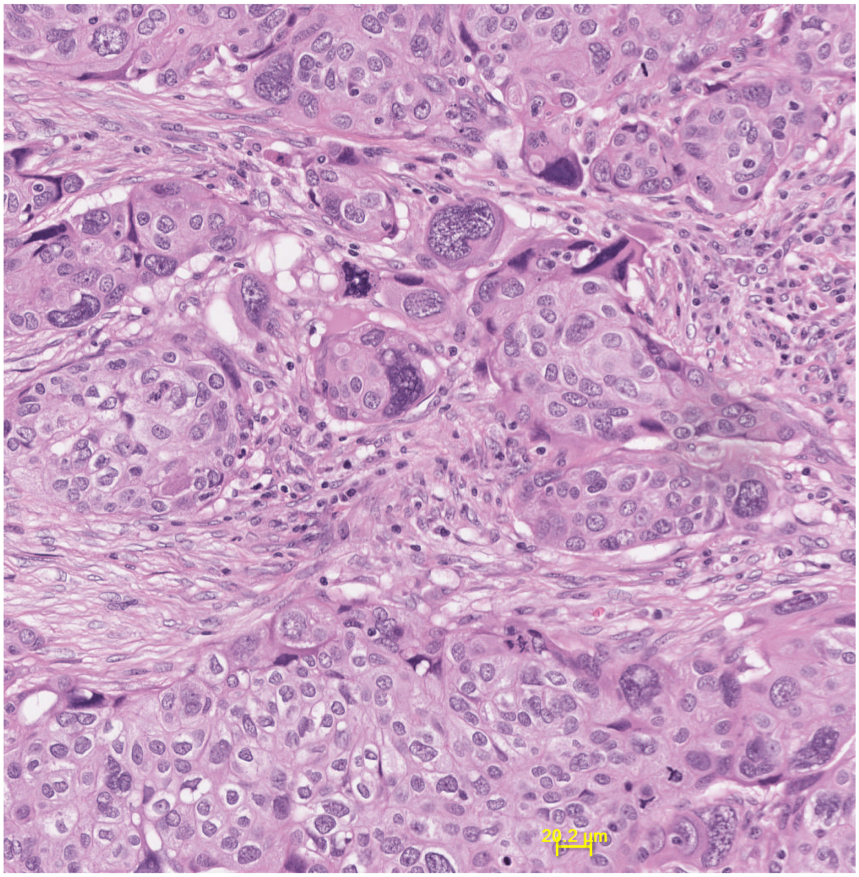

High grade serous carcinoma - Case #4 (treatment-naive)

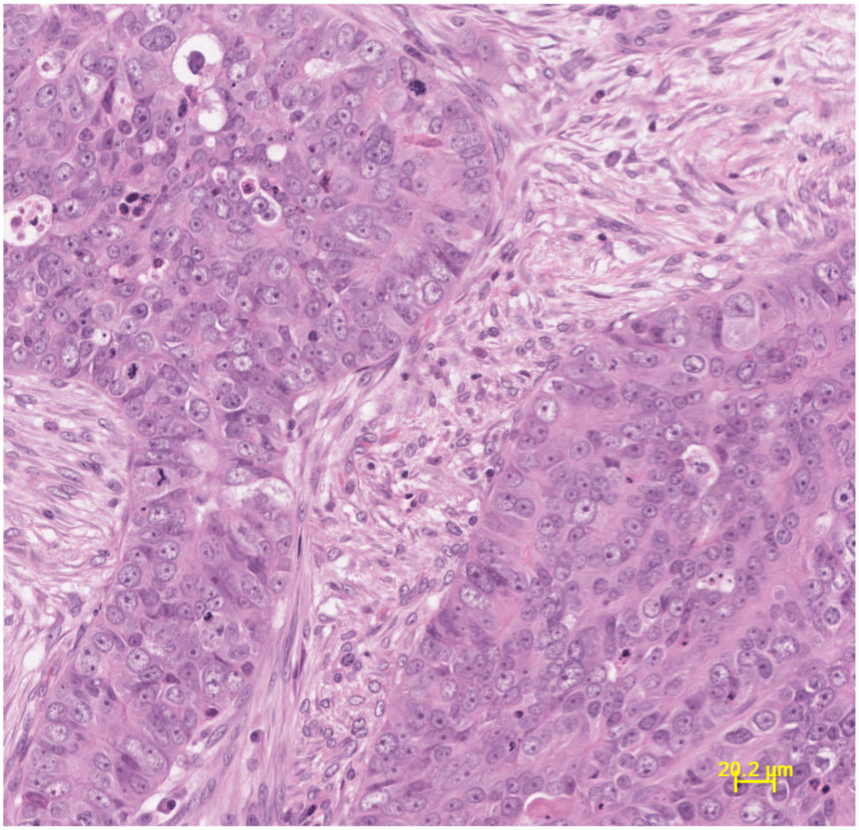

High grade serous carcinoma - Case #5 (treatment-naive)

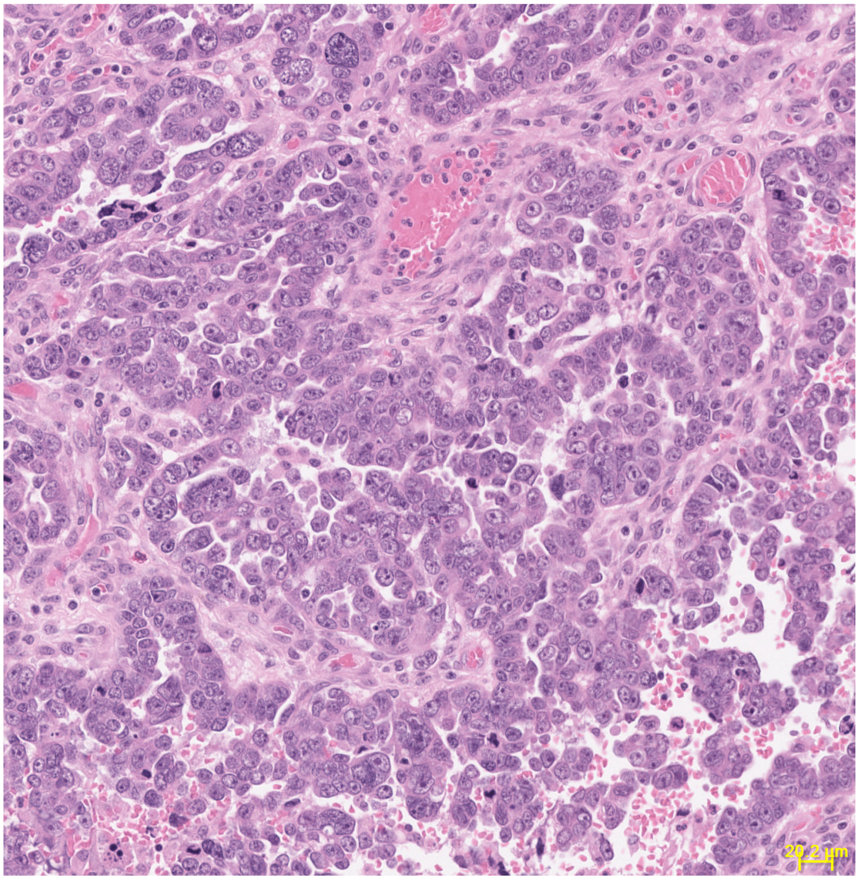

Supplementary Figure 2: Histomorphologic features of high grade serous carcinoma after neo-adjuvant chemotherapy. Case #1-3. Images of patient tumor sections showing high grade serous carcinomas that were treated with neo-adjuvant chemotherapy before their resection. Occasional tumor cells showed marked nuclear enlargement and abnormal lobation or multinucleation, beyond the baseline level of nuclear pleomorphism that was expected for this tumor. Case #4–5. Treatment-naïve high grade serous carcinomas were included for comparison, showing the average tumor with different architectures including papillary fronds, nested and solid areas. Tumor cells showed a moderate level of baseline nuclear atypia, and had prominent nucleoli. Mitotic activity could be seen. Scale bar = 20 µm.

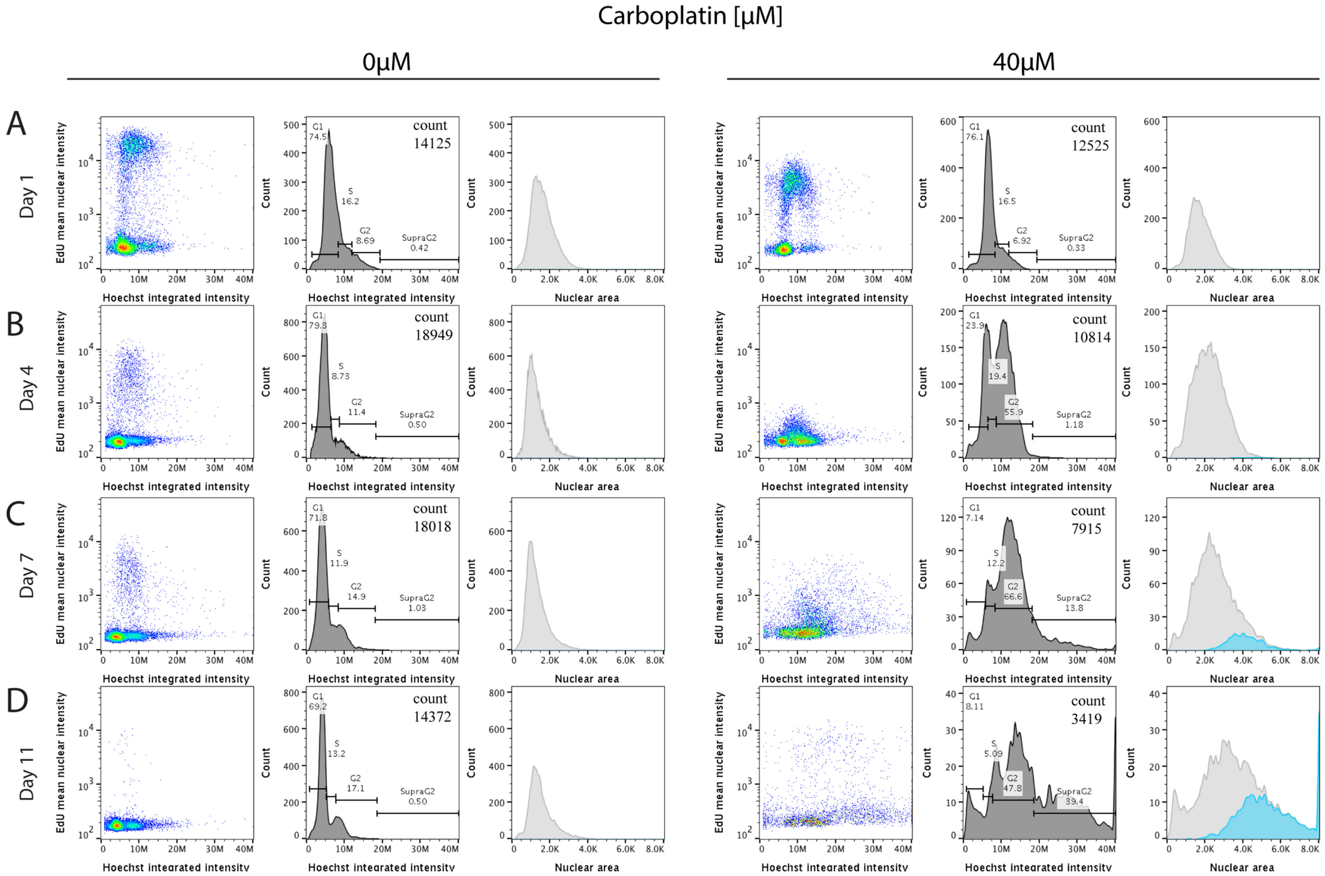

Supplementary Figure 3: Cell cycle gating on the Hoechst DNA ploidy histogram. Adherent OVCAR3 cells maintained within 96-well plates were treated with 0 µM or 40 µM carboplatin for 24 hrs on day 0, followed by removal of drug. Residual cells within the wells on day 1 (**A**), 4 (**B**), 7 (**C**), or 11 (**D**) were then incubated with 10 µM EdU (5-ethynyl-2′-deoxyuridine) for 30 min before fixation, downstream EdU-labeling procedure and Hoechst staining. For each experimental condition, a set of three panels was presented. The left panel shows the 'horseshoe' plot of EdU mean nuclear intensity vs. total Hoechst DNA content. The EdU-high cluster denotes the S-phase subpopulation. The bottom left cluster denotes the G1/(Go) subpopulation whereas the bottom right cluster denotes the G2 subpopulation. The middle panel shows the standard DNA ploidy histogram of total Hoechst DNA content. Gating for G1/(Go), S and G2 were performed based on the position of the respective cluster as shown by the horseshoe plot. A supraG2 subpopulation was also defined to the right of the G2 subpopulation, denoting cells with hyperploid DNA content compared to the baseline ploidy level. The right panel shows histogram of nuclear area where the bulk population was shaded in grey color, and the supraG2 hyperploid subpopulation was overlaid on top in blue color. Data in this figure was representative of three independent experiments. Nuclei count for each condition listed in the middle panel.

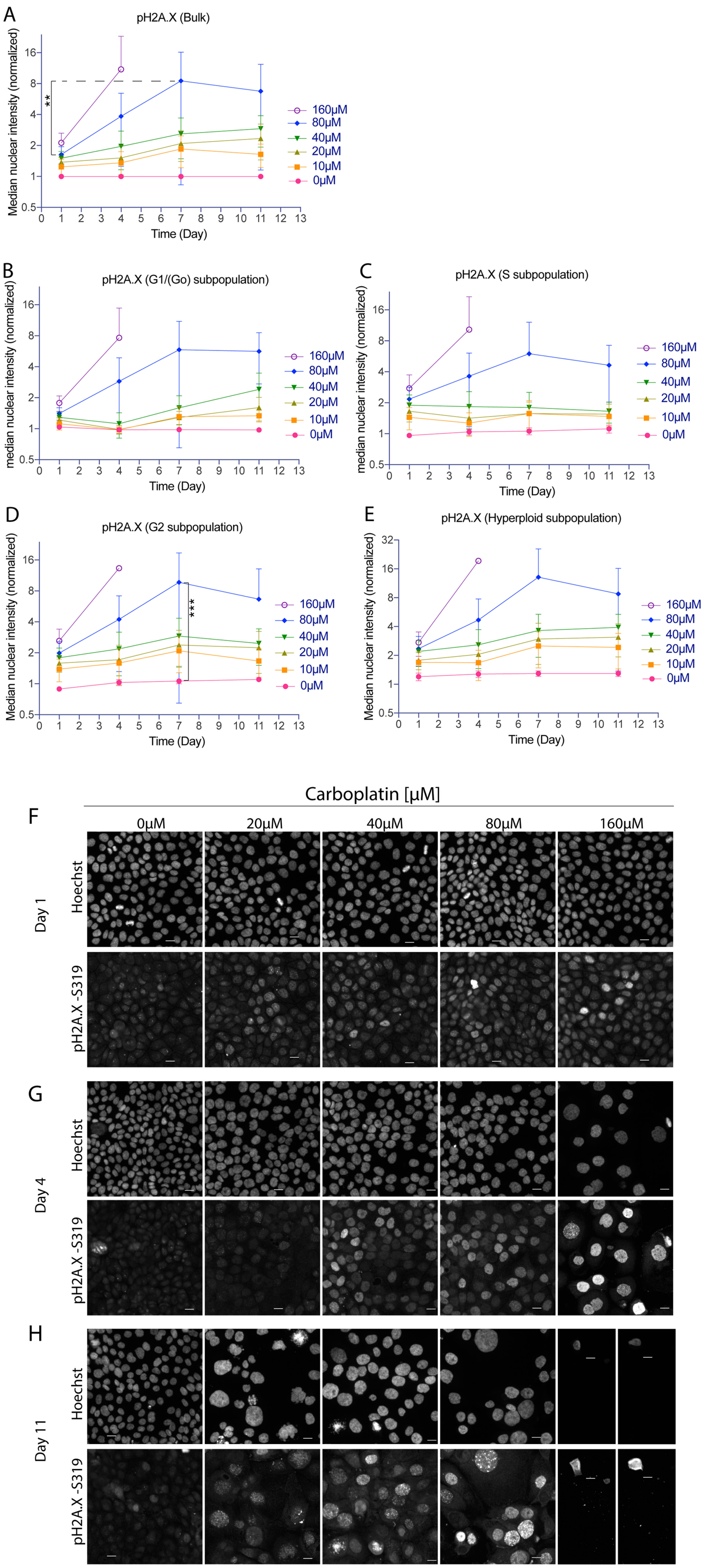
A
pH2A.X (Bulk)
Median nuclear intensity (normalized)
Time (Day)
160μM
80μM
40μM
20μM
10μM
0μM
B
pH2A.X (G1/(Go) subpopulation)
median nuclear intensity (normalized)
C
pH2A.X (S subpopulation)
D
pH2A.X (G2 subpopulation)
E
pH2A.X (Hyperploid subpopulation)
Carboplatin [μM]
F
G
H
0μM
20μM
40μM
80μM
160μM
Day 1
Day 4
Day 11
Hoechst
pH2A.X -S319

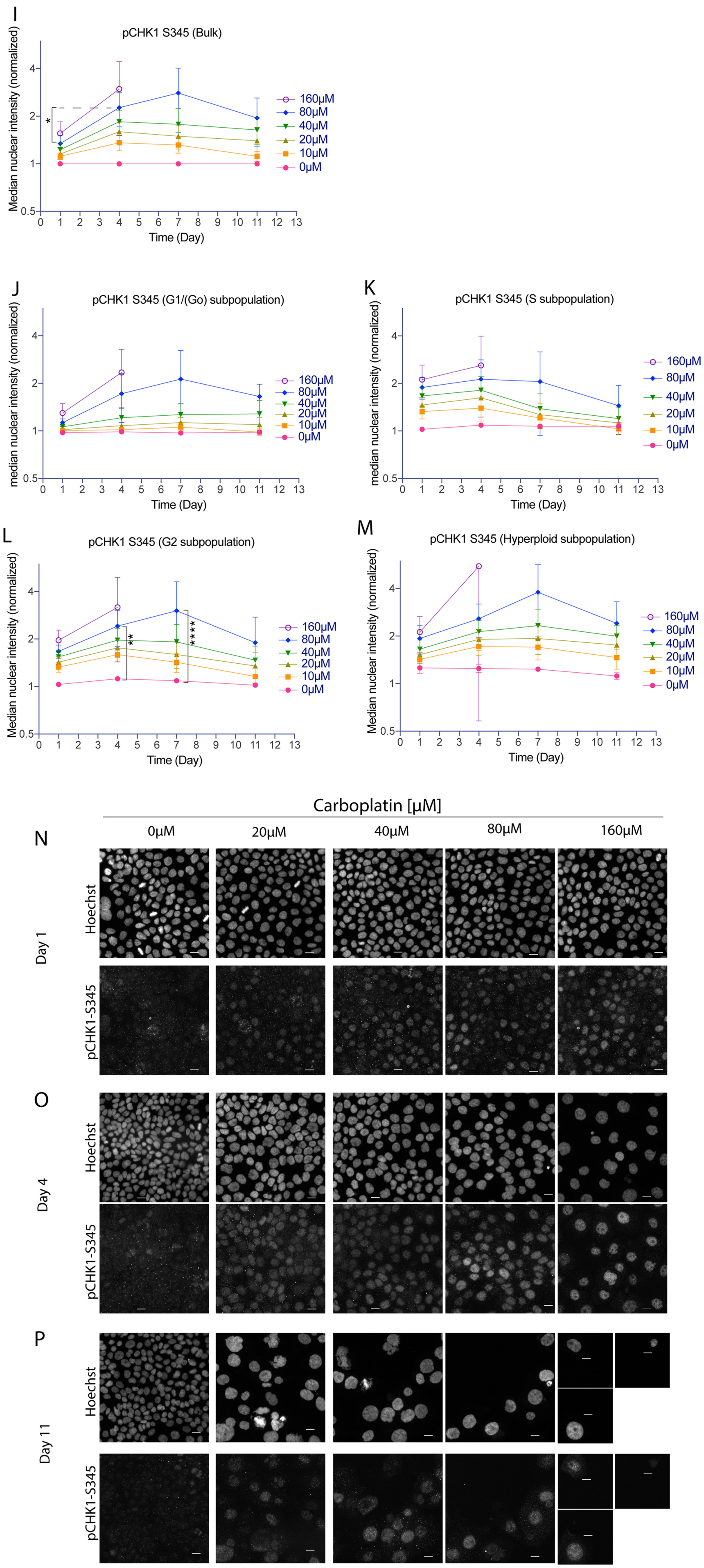
I
pCHK1 S345 (Bulk)
Median nuclear intensity (normalized)
Time (Day)
160µM
80µM
40µM
20µM
10µM
0µM
J
pCHK1 S345 (G1/(Go) subpopulation)
median nuclear intensity (normalized)
K
pCHK1 S345 (S subpopulation)
L
pCHK1 S345 (G2 subpopulation)
M
pCHK1 S345 (Hyperploid subpopulation)
Carboplatin [µM]
0µM
20µM
40µM
80µM
160µM
N
Day 1
Hoechst
pCHK1-S345
O
Day 4
P
Day 11

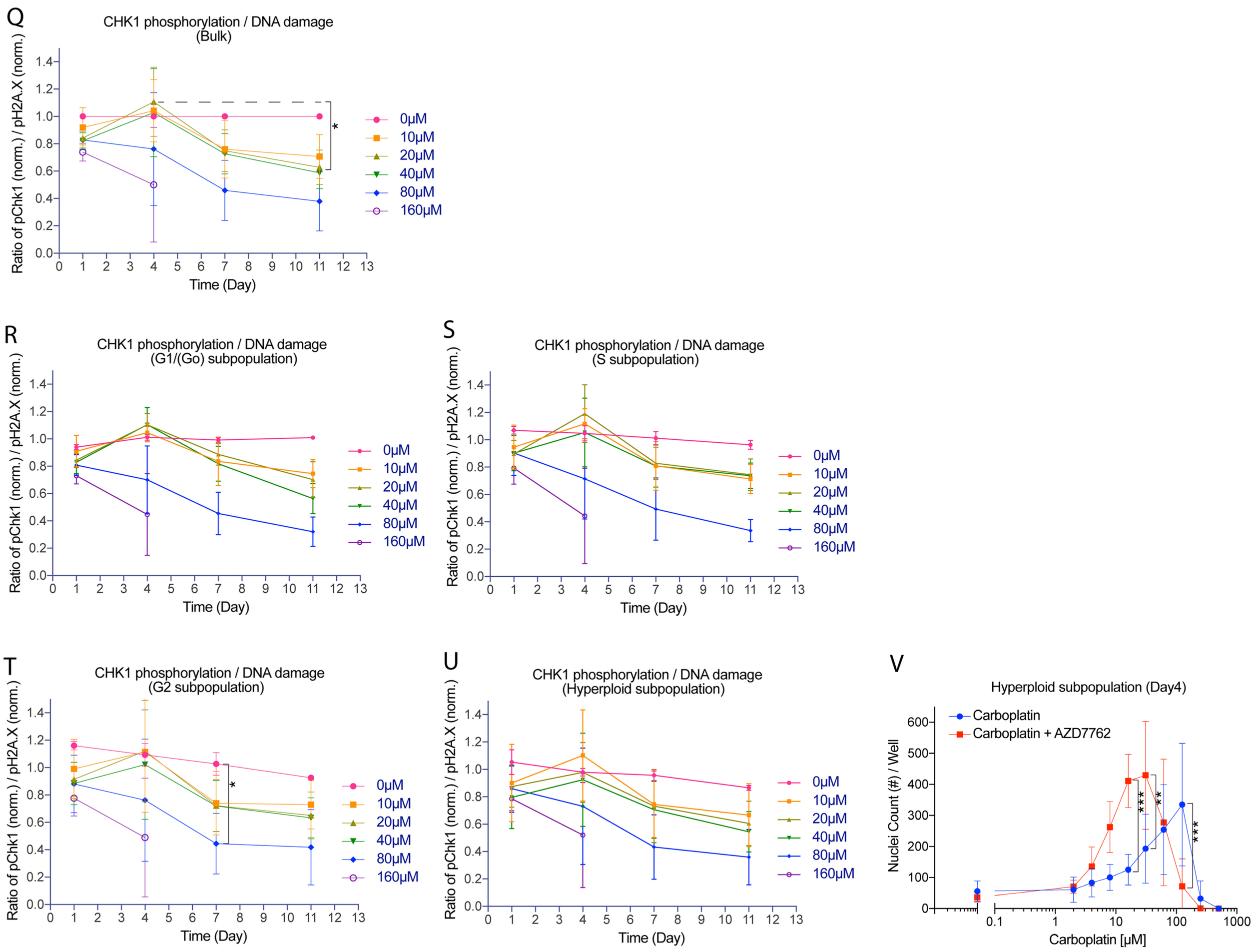

Supplementary Figure 4: DNA damage response and G2/M checkpoint response after carboplatin treatment. Time course experiment where adherent OVCAR3 cells maintained within a set of four similarly prepared 96-well plates were treated with 0 μM to 160 μM carboplatin for 24 hrs on day 0, followed by removal of the drug. The set of four plates were then fixed on day 1, 4, 7 or 11 for immunofluorescence and Hoechst staining of the residual cells within the wells. (**A**–**E**) Time course of DNA damage response as determined from the nuclear staining of H2A.X phospho-serine 319 for the bulk population (A), G1/(Go) (B), S (C), G2 (D) or hyperploid (E) subpopulation. Data points were median nuclear intensity of pH2A.X (normalized to the average value of the bulk control population of each day) +/- standard deviations from 3 independent experiments, plotted on a log-2 scale on the y-axis. Nuclei count ranged from 0 to about 20000 nuclei per well. Wells with fewer than 5 nuclei were not included for analysis. $^{**}p < 0.01$ and $^{***}p < 0.001$ using two-way ANOVA analysis with Bonferroni's correction to demonstrate statistically significant differences for the indicated carboplatin concentration on the same day or for the same treatment concentration between two different time points. (**F**–**H**) Representative images of control or carboplatin-treated cells on day 1 (F), 4 (G), or 11 (H) with Hoechst or pH2A.X staining. Scale bar = 20 μm. (**I**–**M**) Time course of CHEK1 kinase activity as determined from the nuclear staining of CHK1 phospho-serine 345 for the bulk population (I), G1/(Go) (J), S (K), G2 (L) or hyperploid (M) subpopulation. Data points were median nuclear intensity of CHK1 pS345 (normalized to the average value of the bulk control population of each day) +/– standard deviations from 3 independent experiments, plotted on a log-2 scale on the y-axis. Nuclei count ranged from 0 to about 20000 nuclei per well. Wells with fewer than 5 nuclei were not included for analysis. $^{*}p < 0.05$, $^{**}p < 0.01$ and $^{****}p < 0.0001$ using two-way ANOVA analysis with Bonferroni's correction to demonstrate statistically significant differences for the indicated carboplatin concentration on the same day or for the same treatment concentration between two different time points. N-P. Representative images of control or carboplatin-treated cells on day 1 (**N**), 4 (**O**), or 11 (**P**) with Hoechst or pCHK1 staining. Scale bar = 20 μm. (**Q**–**U**) Ratio of normalized pCHK1 to normalized pH2A.X for the bulk population (Q), G1/(Go) (R), S (S), G2 (T), or hyperploid (U) subpopulation. Data points were ratio of normalized pCHK1 / normalized pH2A.X +/– standard deviations from 3 independent experiments. The ratio was also normalized to the bulk control population of each day. Nuclei count ranged from 0 to about 20000 nuclei per well. Wells with fewer than 5 nuclei were not included for analysis. $^{*}p < 0.05$ using two-way ANOVA analysis with Bonferroni's correction to demonstrate statistically significant differences for the indicated carboplatin concentration on the same day or for the same treatment concentration between two different time points. (**V**). Nuclei count of the hyperploid subpopulation on day 4 after initial treatment on day 0 with carboplatin, followed by addition of vehicle or the CHK1 inhibitor AZD-7762 [200 nM] on day 1. Data points were mean nuclei count per well +/– standard deviations from 3 independent experiments. Nuclei analyzed ranged from 0 to about 24000 nuclei per well. $^{**}p < 0.01$ and $^{***}p < 0.001$ using two-way ANOVA analysis with Bonferroni's correction to demonstrate statistically significant differences between carboplatin treatment alone and the combination treatment.

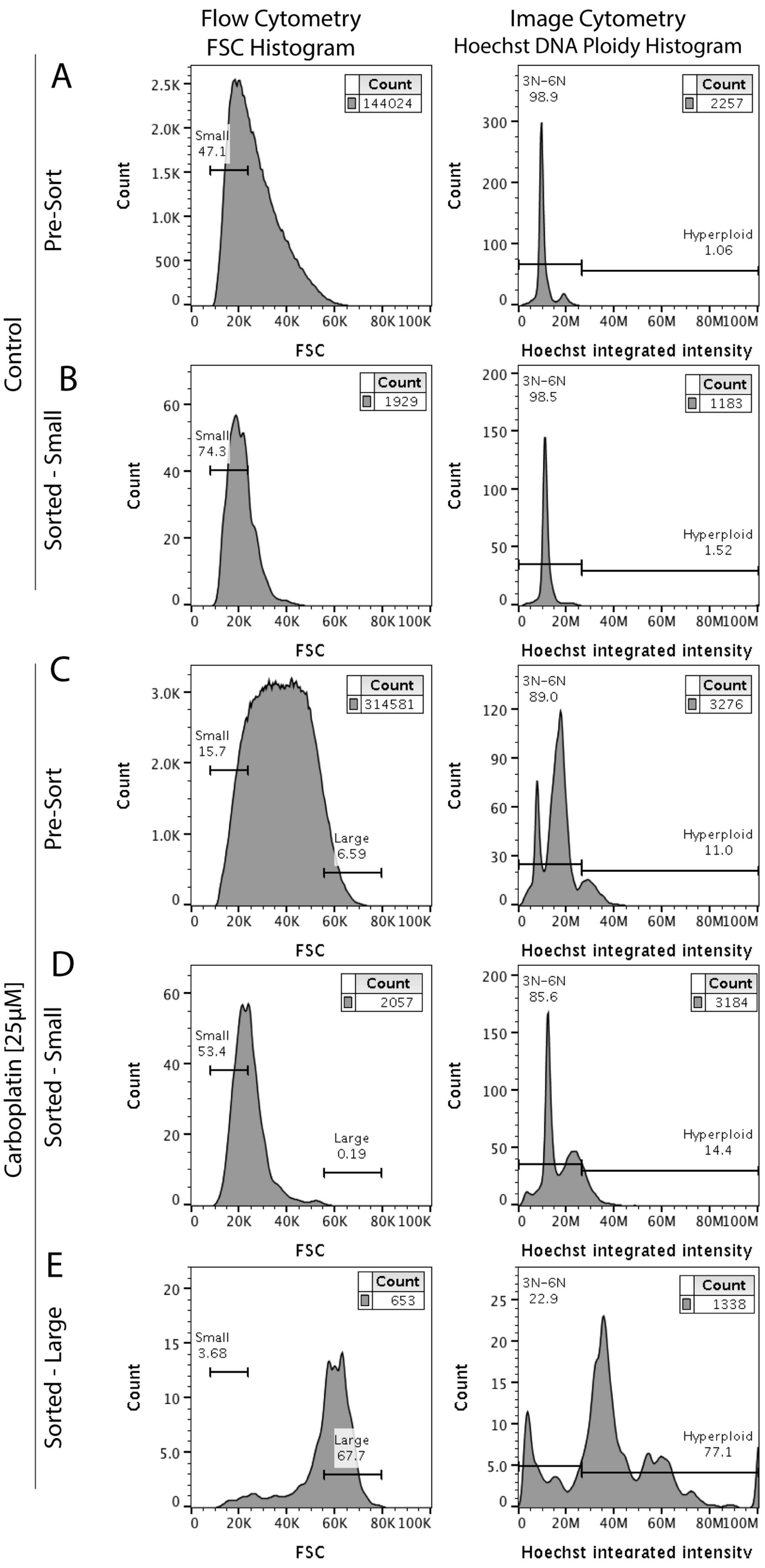
Flow Cytometry
FSC Histogram
Image Cytometry
Hoechst DNA Ploidy Histogram
Control
Carboplatin [25μM]
A
Pre-Sort
Small 47.1
Count 144024
3N–6N 98.9
Count 2257
Hyperploid 1.06
B
Sorted - Small
Small 74.3
Count 1929
3N–6N 98.5
Count 1183
Hyperploid 1.52
C
Pre-Sort
Small 15.7
Large 6.59
Count 314581
3N–6N 89.0
Count 3276
Hyperploid 11.0
D
Sorted - Small
Small 53.4
Large 0.19
Count 2057
3N–6N 85.6
Count 3184
Hyperploid 14.4
E
Sorted - Large
Small 3.68
Large 67.7
Count 653
3N–6N 22.9
Count 1338
Hyperploid 77.1
Count
FSC
Hoechst integrated intensity

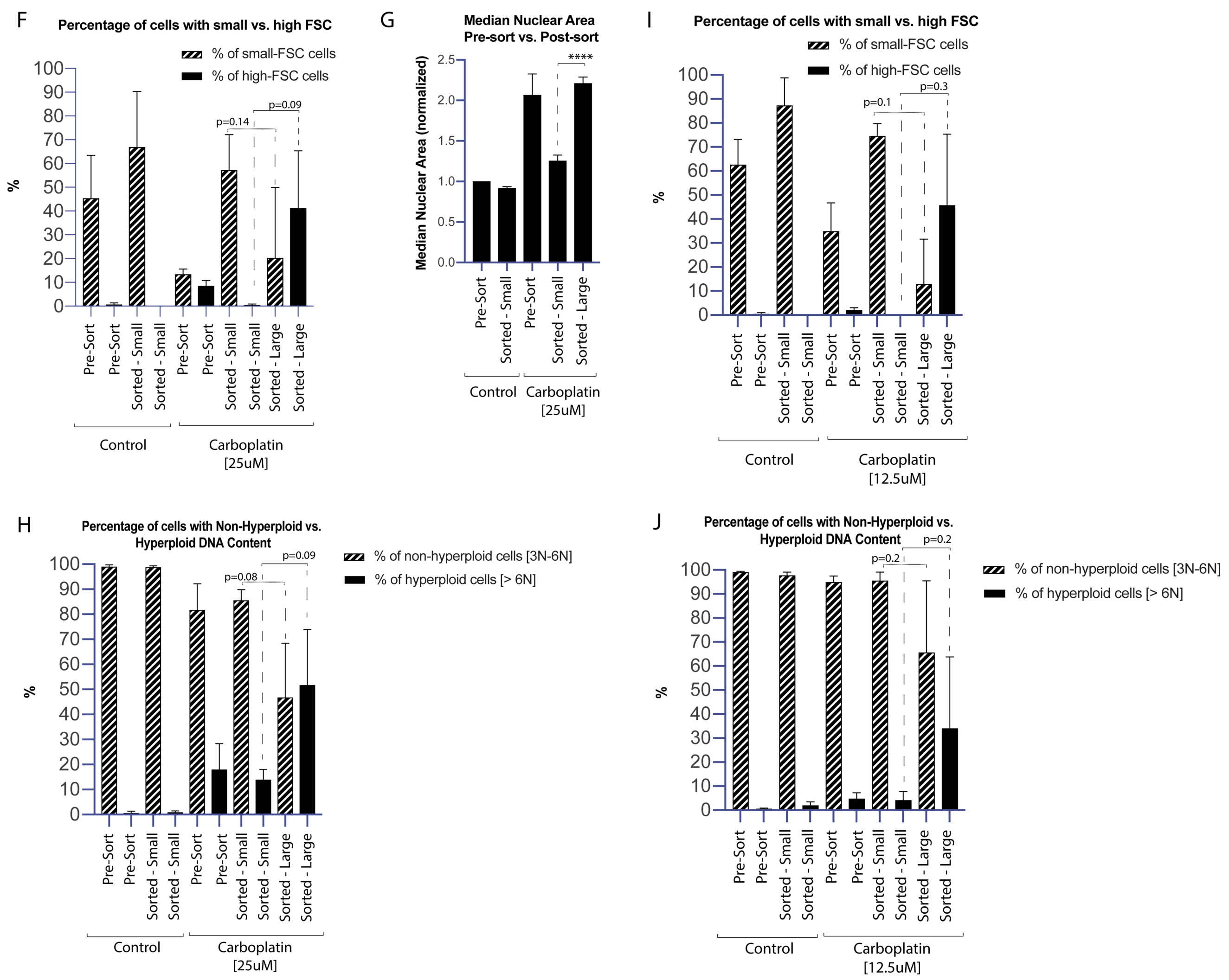

Supplementary Figure 5: Technical setup for flow-sorting by forward scatter and assessment of DNA ploidy status of the sorted samples. Adherent OVCAR3 cells within tissue culture flasks were treated with vehicle, 12.5 µM or 25 µM carboplatin for 24 hrs, followed by removal of the drug and further incubation of the cells for 9 days. Cells were then detached by trypsinization on day 9 and flow sorted by forward scatter and propidium-iodide negativity. The carboplatin-treated cells were sorted into a small and a large subpopulation whereas the control cells were only sorted for a small subpopulation. A portion of the same samples (before sorting or after sorting) were fixed and permeabilized for Hoechst staining to provide the DNA ploidy status by image cytometry analysis. (**A**) The forward scatter (FSC) profile (left panel) of the pre-sort control sample, with a gated region on the left side of the histogram denoting the FSC sorting range for a small-size subpopulation. (**B**) FSC profile (left panel) showing the small-size post-sorted subpopulation from the control sample. C-E. FSC profile (left panels) of the carboplatin-treated sample before (**C**), or after FSC sorting into small (**D**) or large-size (**E**) subpopulation based on the gated region in (C) The right panels in A-E showed the Hoechst DNA ploidy histogram by image cytometry analysis for the same samples before or after sorting. Gated regions on the Hoechst DNA ploidy histogram denote the non-hyperploid (3N-6N) vs. hyperploid (>6N) subpopulation. (**F**) Percent of small-FSC or large-FSC subpopulation in the pre-sort or post-sort, control or carboplatin [25 µM]-treated samples. Data bars were mean percentage +/– standard deviations from 3 independent experiments. Where indicated, *p* values were from two-tailed unpaired *t* test with Welch's correction. (**G**) Median nuclear area of the pre-sort or post-sort, control or carboplatin [25 uM]-treated samples. Data bars were median nuclear area (normalized to the control pre-sort condition) +/– standard deviations from 3 independent experiments. Nuclei analyzed ranged from 250 to 6500 nuclei per well. Where indicated, ****$p < 0.0001$ were from two-tailed unpaired *t* test with Welch's correction. (**H**) Percent of non-hyperploid (3N-6N) or hyperploid (>6N) subpopulation in the pre-sort or post-sort, control or carboplatin [25 µM]-treated samples. Data bars were mean percentage +/– standard deviations from 3 independent experiments. Nuclei analyzed ranged from 250 to 6500 nuclei per well. Where indicated, *p* values were from two-tailed unpaired *t* test with Welch's correction. (**I**) Percent of small-FSC or large-FSC subpopulation in the pre-sort or post-sort, control or carboplatin [12.5 µM]-treated samples. Data bars were mean percentage +/- standard deviations from 3 sorting sessions. Where indicated, *p* values were from two-tailed unpaired *t* test with Welch's correction. (**J**) Percent of non-hyperploid (3N-6N) or hyperploid (>6N) subpopulation in the pre-sort or post-sort, control or carboplatin [12.5 µM]-treated samples. Data bars were mean percentage +/- standard deviations from 3 sorting sessions. Nuclei analyzed ranged from 200 to 4000 nuclei per well. Where indicated, *p* values were from two-tailed unpaired *t* test with Welch's correction.

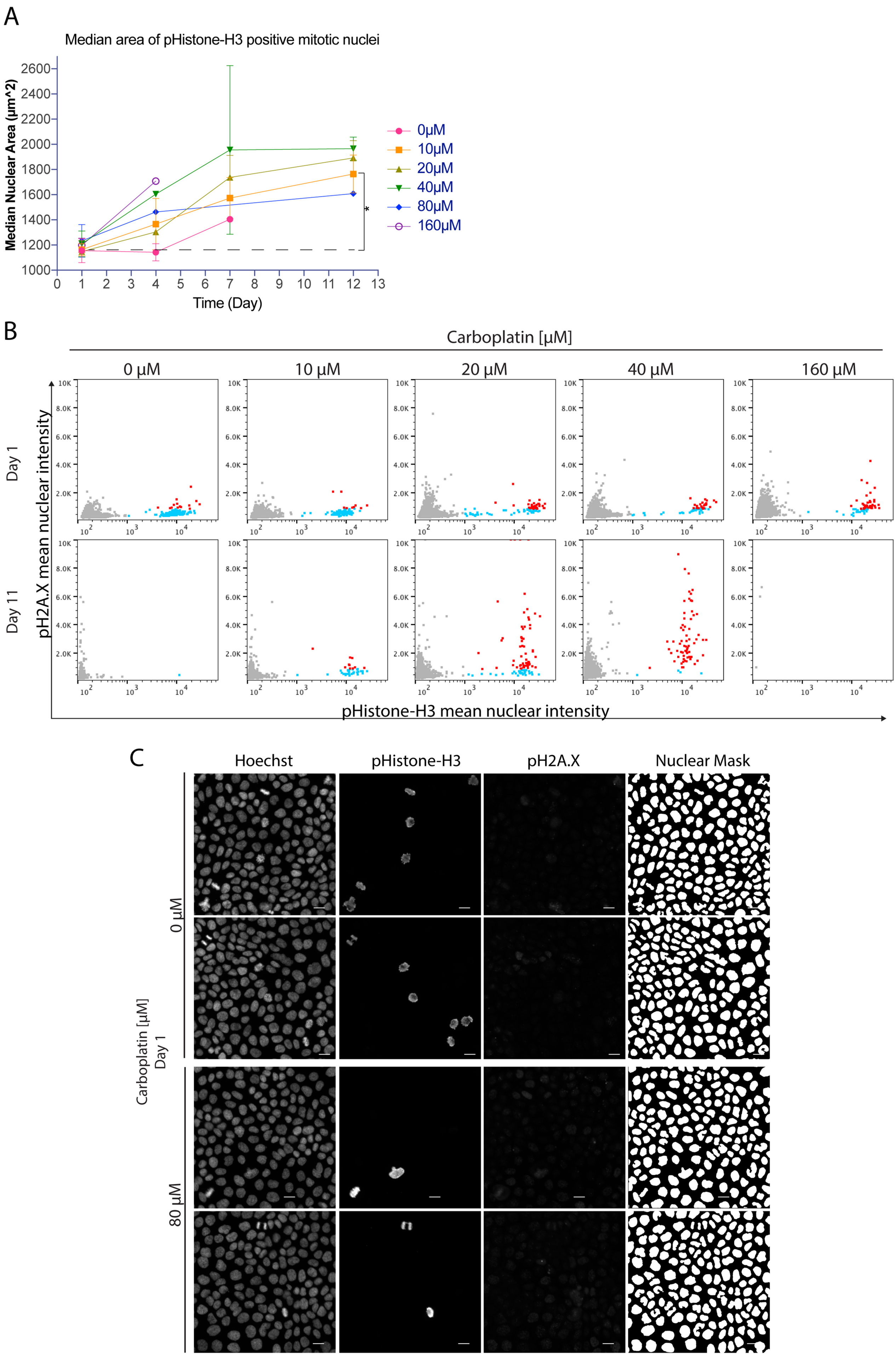
A
Median area of pHistone-H3 positive mitotic nuclei
Median Nuclear Area (µm^2)
Time (Day)
0µM
10µM
20µM
40µM
80µM
160µM
*
B
Carboplatin [µM]
0 µM
10 µM
20 µM
40 µM
160 µM
Day 1
Day 11
pH2A.X mean nuclear intensity
pHistone-H3 mean nuclear intensity
C
Hoechst
pHistone-H3
pH2A.X
Nuclear Mask
0 µM
80 µM
Carboplatin [µM]
Day 1

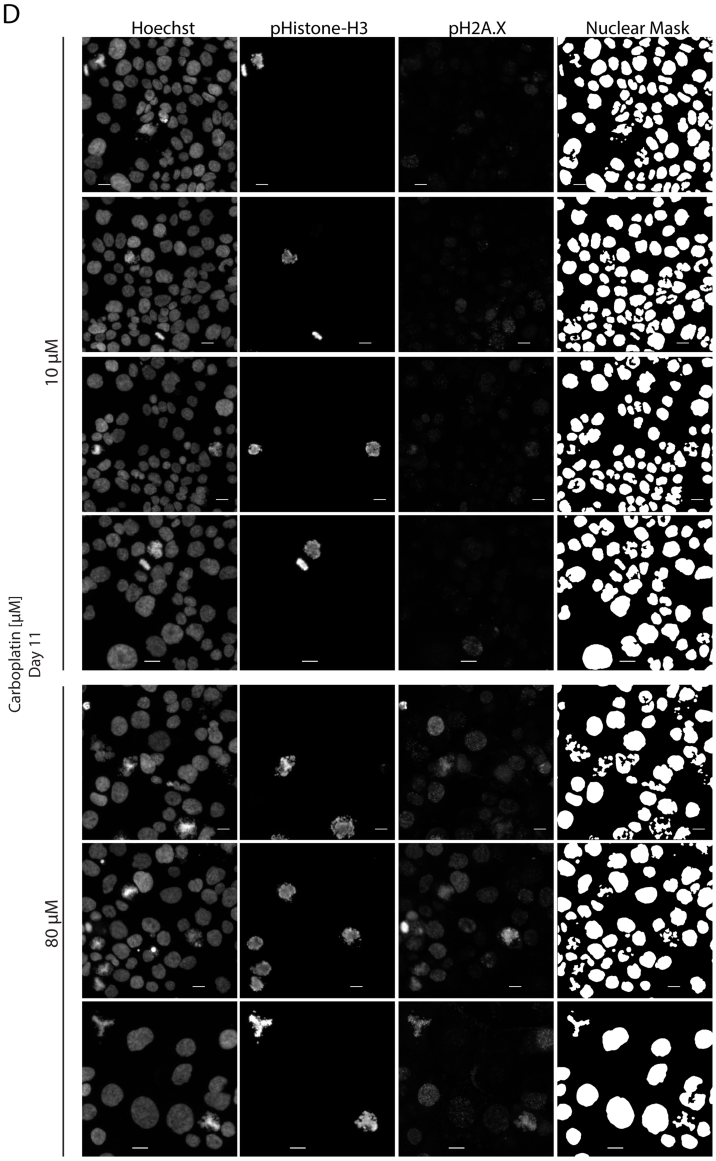

Supplementary Figure 6: Assessment of area and DNA damage on mitotic nuclei. Time course experiment where adherent OVCAR3 cells maintained within a set of four similarly prepared 96-well plates were treated with 0 µM to 160 µM carboplatin for 24 hrs on day 0, followed by removal of the drug. The set of four plates were then fixed on day 1, 4, 7 or 11 for immunofluorescence and Hoechst staining of the residual cells within the wells. (**A**) Median area of pHistone-H3 positive mitotic nuclei on day 1, 4, 7 or 11 after carboplatin treatment. Nuclei count ranged from 1 to about 15000 nuclei per well. Wells with fewer than 5 cells or 5 mitotic events were not included. Values were average of median nuclear area +/- standard deviations from 3 independent experiments. $^{*}p < 0.05$ using two-way ANOVA analysis with Bonferroni's correction to demonstrate statistically significant differences for the 10 µM carboplatin treated condition between two different time points. (**B**) Scatter plot of phospho-Histone H3 mean nuclear intensity (x-axis) plotted against H2A.X pS139 mean nuclear intensity (y-axis) for control or carboplatin-treated cells on day 1 (top panels) or day 11 (bottom panels). Non-mitotic nuclei were shown in grey color and pHistine-H3 positive mitotic nuclei were shown in red or blue color. pH2A.X positivity was defined in the control cells on day 1 with staining intensity higher than the 99.5-percentile level. Red color = pH2A.X positive, blue color = pH2A.X negative. Nuclei count of each condition listed within each panel. (**C**–**D**) Images showing cells on day 1 (C) or day 11 (D) after treatment with 0 µM, 10 µM or 80 µM carboplatin. Mitotic cells were identified by phospho-Histone-H3 staining and the associated level of DNA damage was shown by H2A.X-pS319 co-staining. Control cells were not included for comparison on day 11 as there were no mitotic nuclei identified on day 11 for the control condition. Scale bar = 20 µm. Results in this figure were representative of 3 independent experiments.

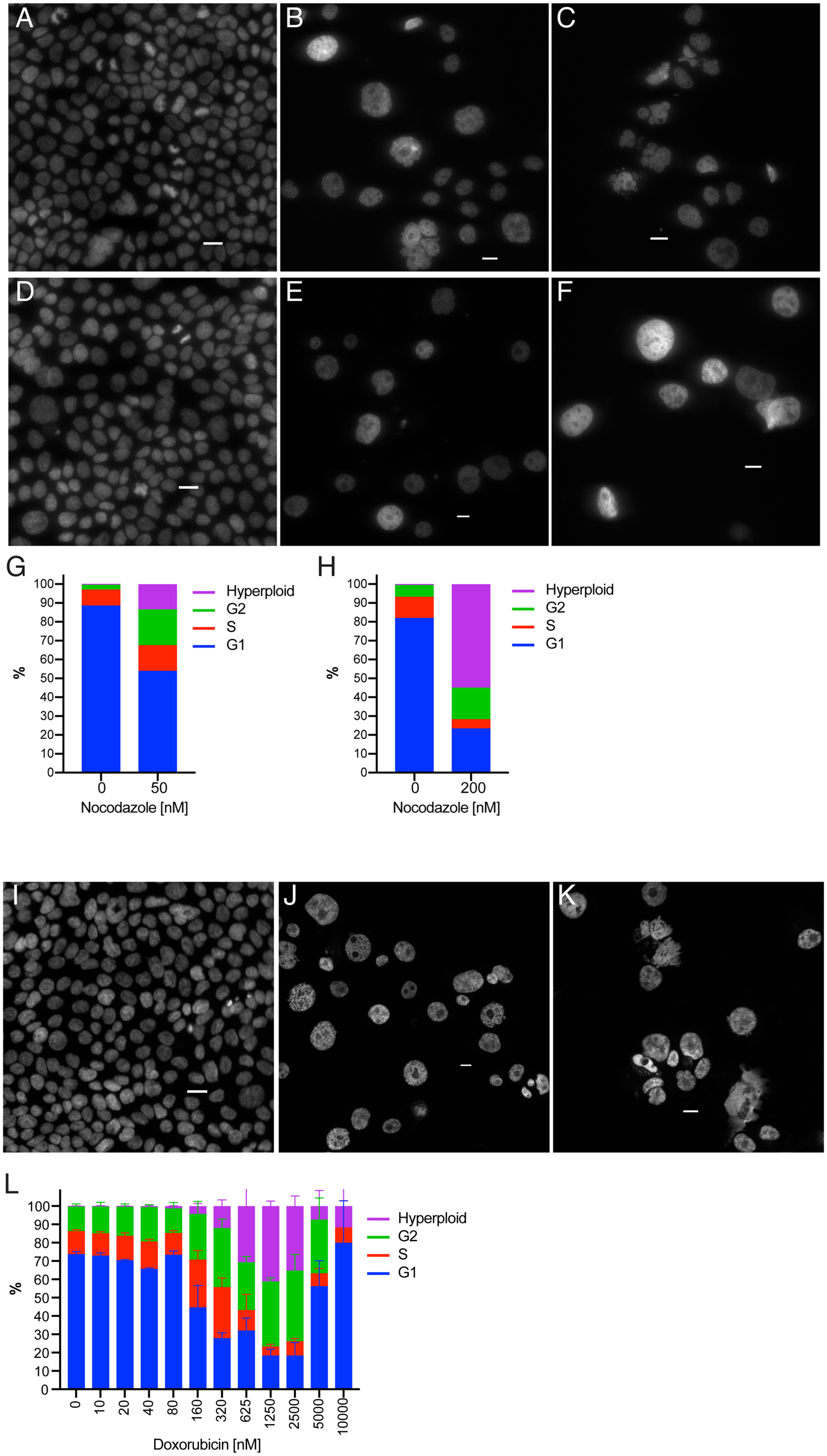
A
B
C
D
E
F
G
H
Hyperploid
G2
S
G1
%
Nocodazole [nM]
0
50
200
I
J
K
L
Doxorubicin [nM]
0
10
20
40
80
160
320
625
1250
2500
5000
10000

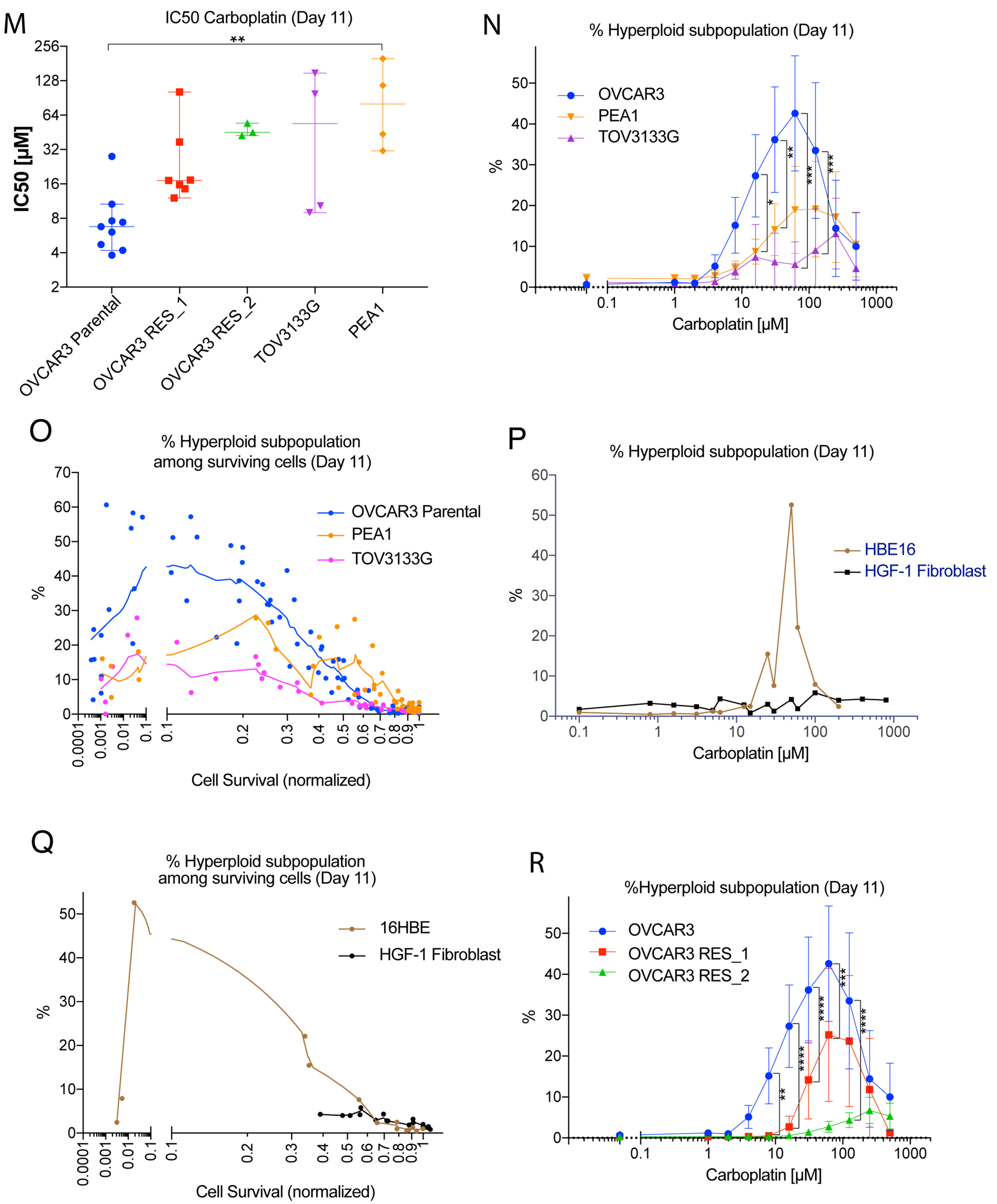
M
IC50 Carboplatin (Day 11)
**
IC50 [μM]
256
128
64
32
16
8
4
2
OVCAR3 Parental
OVCAR3 RES_1
OVCAR3 RES_2
TOV3133G
PEA1
N
% Hyperploid subpopulation (Day 11)
OVCAR3
PEA1
TOV3133G
%
*
**
***
***
Carboplatin [μM]
0.1
1
10
100
1000
O
% Hyperploid subpopulation
among surviving cells (Day 11)
OVCAR3 Parental
PEA1
TOV3133G
%
Cell Survival (normalized)
P
% Hyperploid subpopulation (Day 11)
HBE16
HGF-1 Fibroblast
%
Carboplatin [μM]
Q
% Hyperploid subpopulation
among surviving cells (Day 11)
16HBE
HGF-1 Fibroblast
%
Cell Survival (normalized)
R
%Hyperploid subpopulation (Day 11)
OVCAR3
OVCAR3 RES_1
OVCAR3 RES_2
%
**
****
****
***
****
Carboplatin [μM]

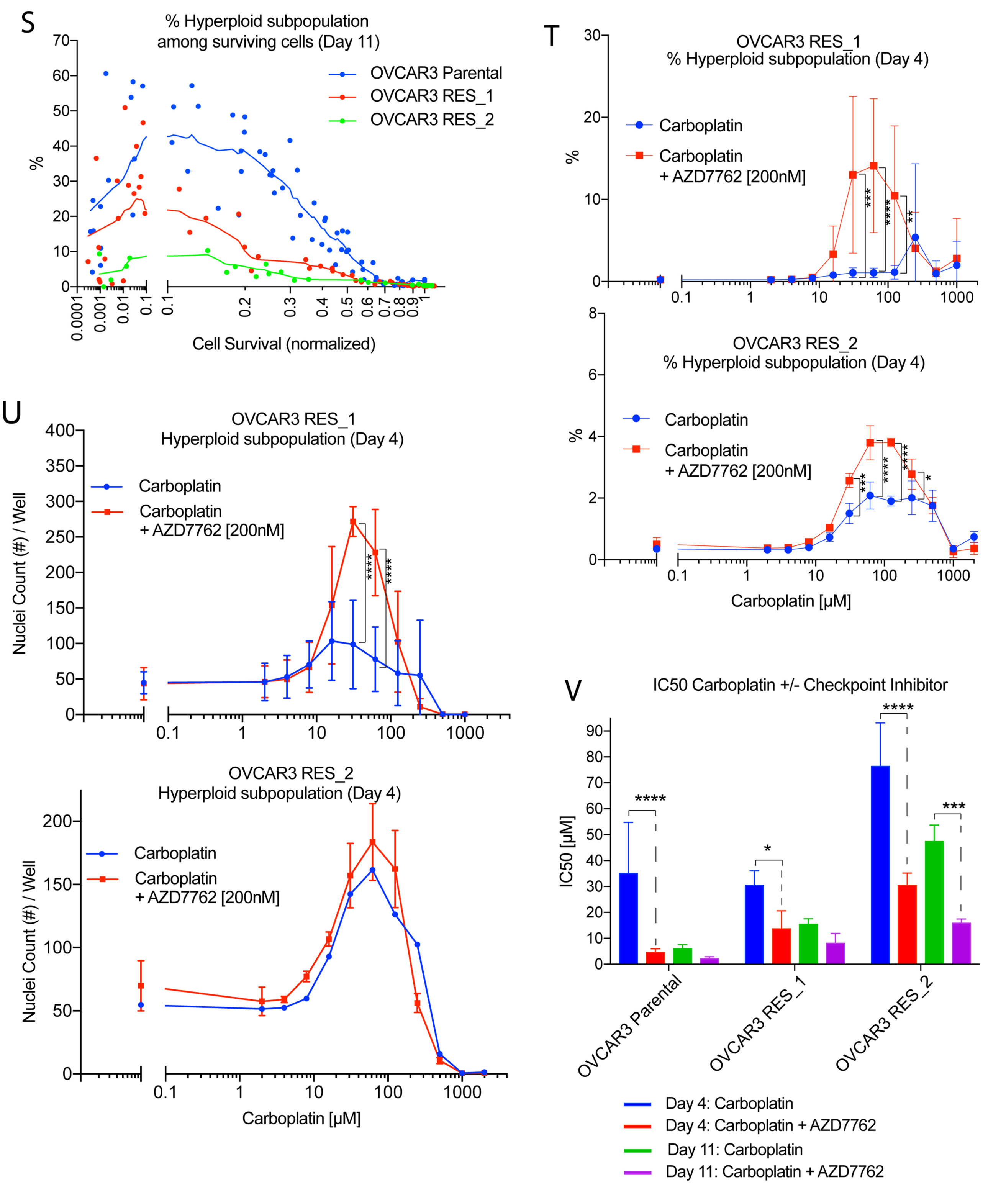

Supplementary Figure 7: Assessment of the hyperploid response across different cell lines and cytotoxic agents and its regulation by CHEK kinase. (**A–H**) OVCAR3 cells treated with vehicle (A, D) or nocodazole at 50 nM (B–C) or 200 nM (E–F) on day 0 continuously for 3 days were then fixed on day 3 and processed for Hoechst staining (A–F). Scale bar = 20 um. (G) Quantification from one experiment showing percentage of cells with the indicated cell cycle/DNA ploidy status by total Hoechst DNA content after vehicle or nocodazole [50 nM] treatment in A–C. 16722 cells from the vehicle-treated well and 811 cells from the nocodazole [50 nM]-treated well were analyzed. (H) Quantification from one experiment showing percentage of cells with the indicated cell cycle / DNA ploidy status by total Hoechst DNA content after vehicle or nocodazole [200 nM] treatment in D–F. 11546 cells from the vehicle-treated well and 204 cells from the nocodazole [200 nM]-treated well were analyzed. (**I–L**) OVCAR3 cells treated with vehicle (I) or doxorubicin at 1.25 uM (J–K) for 24 hrs on day 0, followed by drug removal and then fixation and Hoechst staining on day 11. Scale bar = 20 um. L. Quantification of

cell cycle phase/DNA ploidy distribution by total Hoechst DNA content of OVCAR3 cells on day 11 after 24 hrs of doxorubicin treatment on day 0 with the indicated concentration, followed by drug removal. Nuclei analyzed ranged from 1 to about 20000 nuclei per well. Data bars were the mean percentage of each subpopulation +/– standard deviations from 3 independent experiments. (**M–V**) Carboplatin dose response or combination experiments with checkpoint inhibitor where adherent cells from the different indicated cell lines were treated from 0 μM and up to 1000 μM carboplatin for 24 hrs on day 0, followed by removal of the drug on day 1 and the addition of the CHK1 kinase inhibitor AZD-7762, where indicated. Residual cells within the wells were then analyzed on day 4 or day 11 after fixation and staining. Nuclei analyzed ranged from 0 to about 24000 nuclei per well for OVCAR3 parental, from 0 to about 22000 nuclei per well for OVCAR3 RES_1, from 0 to about 17000 nuclei per well for OVCAR3 RES_2, from 15 to about 27000 nuclei per well for TOV3133G, from 0 to about 6000 nuclei per well for PEA1, from 30 to about 27000 nuclei per well for 16HBE, and from 460 to about 4000 nuclei per well for HGF-1. M. The IC50 value for carboplatin cytotoxicity based on twelve-point dose response evaluation. The median IC50 values +/– standard deviations from at least 3 independent experiments, along with the value from each experiment, were plotted on a log-2 scale. $^{**}p < 0.01$ using two-way ANOVA analysis with Bonferroni's correction to demonstrate statistically significant differences for the two cell lines. The TOV3133G and PEA1 cell line appeared to exhibit some level of relative platinum resistance as compared to the OVCAR3 parental cell line. Two carboplatin-resistant daughter cell lines were generated *in-vitro* from the parental OVCAR3 cell line on separate occasions after several discrete cycles of carboplatin treatment (see Methods and Materials for details). The resistant daughter cell lines OVCAR3 RES1 and RES2 exhibited 2.5-fold and 6.7-fold higher platinum-resistance, respectively, as compared to the parental cell line. (N, P, R) Carboplatin concentration-dependent effect on percentage of hyperploid subpopulation on day 11 for three different ovarian cancer cell lines (OVCAR3 parental, TOV3133G, PEA1) (N), the SV40 large-T antigen transformed lung epithelial (16HBE) and the non-transformed gingival fibroblast (HGF-1) cell line (P), or for the OVCAR3 parental (re-plotted) and two *in-vitro* generated carboplatin-resistant daughter cell lines (OVCAR3 RES_1, RES_2) (R). Values were mean percentage +/– standard deviation from 3 independent experiments for N and R, or percent values from two independent experiments pooled together for P. $^{*}p < 0.05$, $^{**}p < 0.01$, $^{***}p < 0.001$ and $^{****}p < 0.0001$ using two-way ANOVA analysis with Bonferroni's correction to demonstrate statistically significant differences for the indicated carboplatin concentration between the different cell lines. (O, Q, S) Percentage of hyperploid subpopulation on day 11 plotted against cell survival to demonstrate the *hyperploid response* for the OVCAR3 parental, TOV3133G, PEA1 cell line (O), the 16HBE and HGF-1 cell line (Q), or the OVCAR3 parental (re-plotted) and two matched carboplatin-resistant daughter cell lines (OVCAR3 RES_1 and RES_2) (S). Cell survival was normalized to the control condition of each cell line. Data points were from three (O, S) or two (Q) independent experiments pooled together. A best-fit line was plotted using the LOWESS function in Prism for each cell line. The OVCAR3 parental and the 16HBE transformed lung epithelial cell line showed a substantial hyperploid response whereas the PEA1 cell line showed an intermediate level and the TOV3133G cell line showed a low level of this hyperploid response, similar to that exhibited by the OVCAR3 carboplatin-resistant daughter cell lines (OVCAR3 RES_1, RES_2). The HGF-1 fibroblast cell line showed no significant hyperploid response. T-U. Percentage (T) or nuclei count (U) of the hyperploid subpopulation in the OVCAR3 resistant daughter cell lines RES_1 (top panel) and RES_2 (bottom panel) on day 4 after treatment with carboplatin or carboplatin + CHK1 inhibitor AZD-7762 [200 nM]. Data points were mean percentage of hyperploid subpopulation +/– standard deviations from 3 independent experiments. $^{*}p < 0.05$, $^{**}p < 0.01$, $^{***}p < 0.001$ and $^{****}p < 0.0001$ using two-way ANOVA analysis with Bonferroni's correction to demonstrate statistically significant differences between carboplatin treatment alone and the combination treatment. The incubation of carboplatin-treated cells with the CHEK1 kinase inhibitor AZD-7762 resulted in a substantial increase in the hyperploid subpopulation on day 4 when tumor cells were otherwise still arrested in the G2-phase. V. IC50 value for OVCAR3 parental and matched carboplatin-resistant daughter cell lines (OVCAR3 RES_1 and RES_2) after treatment with carboplatin on day 0, followed by removal of the drug on day 1 and the addition of vehicle or AZD-7762 [200 nM]. $^{*}p < 0.05$, $^{***}p < 0.001$ and $^{****}p < 0.0001$ using two-way ANOVA analysis with Bonferroni's correction to demonstrate statistically significant differences between the indicated treatment conditions. Treatment with AZD-7762 to inhibit the G2-M checkpoint rendered the OVCAR3 resistant cell lines 2 to 3-fold more sensitive to carboplatin treatment.
